# Consensus Paper: Towards a Systems-Level View of Cerebellar Function: the Interplay Between Cerebellum, Basal Ganglia, and Cortex

**DOI:** 10.1007/s12311-016-0763-3

**Published:** 2016-02-13

**Authors:** Daniele Caligiore, Giovanni Pezzulo, Gianluca Baldassarre, Andreea C. Bostan, Peter L. Strick, Kenji Doya, Rick C. Helmich, Michiel Dirkx, James Houk, Henrik Jörntell, Angel Lago-Rodriguez, Joseph M. Galea, R. Chris Miall, Traian Popa, Asha Kishore, Paul F. M. J. Verschure, Riccardo Zucca, Ivan Herreros

**Affiliations:** 1 Istituto di Scienze e Tecnologie della Cognizione, Consiglio Nazionale delle Ricerche (ISTC-CNR), Via San Martino della Battaglia 44, 00185 Rome, Italy; 2 Systems Neuroscience Institute, Department of Neurobiology, and Center for the Neural Basis of Cognition, University of Pittsburgh, 3501 Fifth Avenue, 4079 BST-3, Pittsburgh, PA 15261 USA; 3 Neural Computation Unit, Okinawa Institute of Science and Technology, 1919-1 Tancha, Onna-son, Kunigami-gun, Okinawa 904-0495 Japan; 4 Department of Neurology, Radboud University Nijmegen Medical Center (HP 935), PO Box 9101, 6500 HB Nijmegen, The Netherlands; 5 Department of Physiology, Northwestern University Feinberg School of Medicine, 303 East Chicago Avenue M211, Chicago, IL 60611 USA; 6 Neural Basis of Sensorimotor Control, Department of Experimental Medical Science, Lund University, BMC F10 Tornavägen 10, 221 84 Lund, Sweden; 7 School of Psychology, University of Birmingham, Edgbaston, Birmingham B15 2TT UK; 8 Human Motor Control Section, National Institute of Neurological Diseases and Stroke (NINDS), National Institutes of Health (NIH), Bethesda, MD 20982 USA; 9 Comprehensive Care Centre for Movement Disorders, Sree Chitra Tirunal Institute for Medical Sciences and Technology, Kerala, 695011 India; 10 Department of Information and Communication Technologies, Universitat Pompeu Fabra, Barcelona, Spain; 11 Catalan Institute of Research and Advanced Studies (ICREA), Barcelona, Spain

**Keywords:** Basal ganglia cerebellum anatomical link, Nucleo-olivary inhibition, Movement disorders, Parkinson’s disease tremor, Cerebellar motor and cognitive function, Non-invasive brain stimulation

## Abstract

Despite increasing evidence suggesting the cerebellum works in concert with the cortex and basal ganglia, the nature of the reciprocal interactions between these three brain regions remains unclear. This consensus paper gathers diverse recent views on a variety of important roles played by the cerebellum within the cerebello-basal ganglia-thalamo-cortical system across a range of motor and cognitive functions. The paper includes theoretical and empirical contributions, which cover the following topics: recent evidence supporting the dynamical interplay between cerebellum, basal ganglia, and cortical areas in humans and other animals; theoretical neuroscience perspectives and empirical evidence on the reciprocal influences between cerebellum, basal ganglia, and cortex in learning and control processes; and data suggesting possible roles of the cerebellum in basal ganglia movement disorders. Although starting from different backgrounds and dealing with different topics, all the contributors agree that viewing the cerebellum, basal ganglia, and cortex as an integrated system enables us to understand the function of these areas in radically different ways. In addition, there is unanimous consensus between the authors that future experimental and computational work is needed to understand the function of cerebellar-basal ganglia circuitry in both motor and non-motor functions. The paper reports the most advanced perspectives on the role of the cerebellum within the cerebello-basal ganglia-thalamo-cortical system and illustrates other elements of consensus as well as disagreements and open questions in the field.

## Introduction

The cerebellum works in concert with cortex and the basal ganglia as a fundamental building block in motor and cognitive tasks of various complexity, from sensorimotor mapping to reasoning [1–4]. This *systems-level view* is increasingly supported by evidence demonstrating that the cerebellum and the basal ganglia receive input from, and send output to, different cortical areas through multisynaptic loops that have been assumed to be anatomically segregated and to perform distinct functional operations [5–10]. Moreover, recent findings reveal the existence of an anatomical substrate for the bidirectional communication between cerebellum and basal ganglia. In particular, studies on rats [11] and monkeys [12] have demonstrated that the cerebellum sends a strong disynaptic projection to the striatum through the thalamus. Furthermore, recent studies in monkeys have shown that the subthalamic nucleus sends a disynaptic projection to the cerebellar cortex by way of the pontine nuclei [13]. Similar evidence has been recently reported in the human brain [14].

These data have stimulated new research to investigate the reciprocal influence between these brain areas and the different forms of learning typically associated with them: supervised learning in the cerebellum based on plasticity of parallel fiber-Purkinje cell synapses [15–17]; unsupervised learning in the cortex based on associative Hebbian processes [18–20]; and trial-and-error (reinforcement learning) in the basal ganglia based on reward prediction errors computed at level of the striatum and depending on dopamine signals [21, 22]. Several recent studies have tried to integrate these apparently disconnected learning processes, focusing for example on the role of the cerebellum in regulating the plasticity of premotor and motor networks during sensorimotor learning [23–27]; the involvement of the cerebellum in the reinforcement learning processes underlying the computation of sensory and reward prediction errors during motor adaptation [28–33]; the role of the cerebellum and basal ganglia in the cortical acquisition of high-level cognitive (non-motor) functions [1, 3, 4]; and the influence of cerebellum and basal ganglia on motor cortical plasticity in health and in Parkinson’s disease [34, 35]. Finally, data supporting a close interaction between cerebellar, basal ganglia, and cortical areas have recently stimulated new research on the role of cerebellum and basal ganglia in functions typically associated with cortical areas (e.g., action understanding [4]) and the role of cerebellar and cortical areas in impairments typically associated with basal ganglia, such as Tourette’s syndrome, dystonia, and Parkinson’s disease [36–40].

Despite this recent progress, we still lack a comprehensive framework that defines cerebellar function from a wider, systems-level perspective [4, 41, 42]. Viewing cerebellum, cortex, and basal ganglia as an integrated system could lead to understand their functional and learning processes in radically different ways. The goal of this consensus paper is to feed the discussion on the *fundamental interactions between the functional and plasticity processes of cerebellum, cortex, and basal ganglia, conceived as forming an integrated system underlying several motor and cognitive functions.*

Evidence of the interactions between the functional and learning processes in the cerebellum, cortex, and basal ganglia comes from different scientific disciplines and methodologies: from neurophysiology to computational modelling and from behavioral methods to neuropsychological and brain imaging approaches. Unfortunately, discussion between experts using these different approaches is often limited. We believe that a multi-methodological and multidisciplinary discussion is necessary to fully understand the integrated dynamics of the cerebellar-basal-ganglia-cortical system. This consensus paper offers an up-to-date overview on this discussion as it collects contributions from an interdisciplinary community of scientists actively involved in the investigation of cerebellum that provide a range of different, sometimes controversial, viewpoints.

The issue begins with the paper of Bostan and Strick that introduces and reviews the recent neurophysiological and neuroanatomical evidence on the cerebellar connections with the cerebral cortex and the basal ganglia.

The next two contributions, by Verschure et al. and by Jörntell, further expand the systems-level view of cerebellar interconnections supported by the analysis of the previous piece (by Bostan and Strick). Verschure et al. raise the fascinating possibility that the nucleo-olivary pathway balances the contribution of feedback (mediated by brain stem nuclei) and feed-forward (cerebellar) modes of motor control. Jörntell provides an overview of the spinal inputs into the cerebellum, discussing how these inputs allow the cerebellum to keep informed about the excitatory drive on low level motor functions. He thus proposes that the major role of the cerebellum is to allow many spinal inputs to be associated with one another in specific environmental contexts.

The contribution of Houk provides a theoretical, systems-level perspective of how the cerebellar-cortical and basal ganglia-cortical loops may interact to affect learning and control processes.

Along similar lines, the contribution of Doya elaborates his previous theory of motor control and learning, based on the idea that different brain areas implement different learning algorithms, to account for recent findings discussed by Bostan and Strick on the circuitry between the cerebellum and the basal ganglia.

Pivoting on recent empirical evidence, Miall suggests that the cerebellum may support a single operation, acting as a short-term predictor within the cerebellar-basal-ganglia-cortical system. He also discusses data on medium-term, post-learning, memory consolidation effects in this system.

Lago-Rodriguez and Galea summarize the importance of using non-invasive techniques (such as transcranial magnetic stimulation (TMS) or transcranial direct current stimulation (tDCS)) to investigate the causal role of the cerebellum in motor function and its interactions with other brain regions.

Finally, the last two papers provide some examples of the consequences of abnormal interactions between cerebellum, basal ganglia, and cortical systems. Popa and Kishore discuss data supporting how impaired cerebellar plasticity mechanisms could contribute to Parkinson’s disease and dyskinesia. Caligiore et al. build on recent data that suggest an involvement of cerebellum in Parkinson’s disease and discuss some cortical-subcortical circuits within the cerebellar-basal-ganglia-cortical network that could be involved in Parkinson’s resting tremor.

Taken together, these empirical and theoretical contributions offer readers a broad and up-to-date framework to describe the dynamic interplay between functioning and learning mechanisms in the cerebellum, basal ganglia, and cortex, pointing out the key open issues for future research. The final part of the article summarizes the main topics and analyzes the main elements of consensus—or lack of consensus among the authors. As discussed in the “Summary and conclusions” section, consensus exists on several key aspects of cerebellar-basal-ganglia-cortical function; at the same time, there are various topics that are still debated and where consensus cannot be reached yet, reflecting the relative novelty of the topic. These open points represent interesting and promising directions for future research.

## Cerebellar Connections with the Cerebral Cortex and the Basal Ganglia (A.C. Bostan, P.L. Strick)

The classical view of cerebellar function has been that it is exclusively concerned with the control of movement through descending control and influence on the primary motor cortex (M1). The cerebellum was thought to receive information from multiple neocortical areas and funnel it back to M1 [43]. More recent analyses of cerebellar outputs have resulted in a dramatic shift in the conceptualization of cerebellar function.

**Fig. 1:**
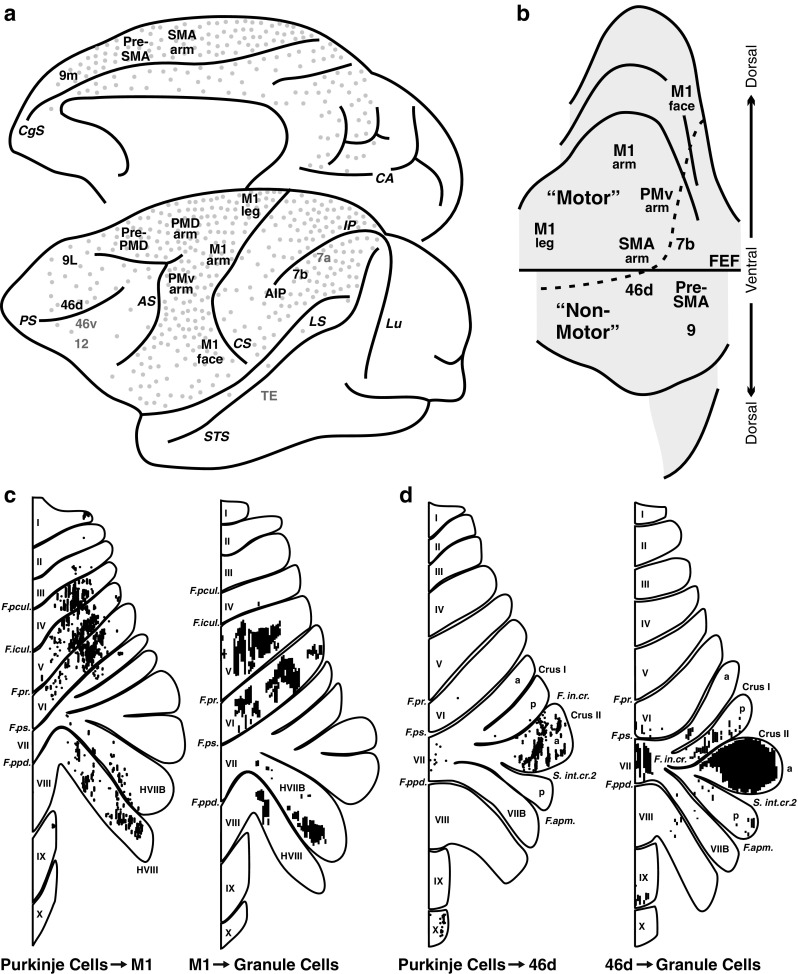
Cerebellar networks with the cerebral cortex. **a** The relative density of cerebral cortex neurons that project to the pontine nuclei is indicated by *gray dots* on the lateral and medial views of the monkey brain (based on [48]). *Black labels* indicate areas of the cerebral cortex that are the target of cerebellar output. *Gray labels* indicate areas that are not targets of cerebellar output (adapted from [3]). **b** Summary map of dentate nucleus output topography. The *lettering* on the unfolded map indicates the neocortical target of different output channels. The location of different output channels divides the dentate nucleus into motor and non-motor domains, separated by the *dotted line* (adapted from [45]). **c** Organization of cerebellar circuits with M1. *Left*: the distribution of Purkinje cells (*small dots*) that project to the arm area of M1. *Right*: the distribution of granule cells (*fine lines*) that receive input from the arm area of M1 (adapted from [50]). **d** Organization of cerebellar loops with area 46. *Left*: the distribution of Purkinje cells (*small dots*) that project to area 46. *Right*: the distribution of granule cells (*fine lines*) that receive input from area 46 (adapted from [50]). *Numbers* refer to cytoarchitectonic areas (**a**, **b**). *Roman numerals* refer to cerebellar lobules (**c**, **d**). *Labels in italics* refer to cortical sulci (**a**) and cerebellar fissures (**c**, **d**). *AIP* anterior intraparietal area, *AS* arcuate sulcus, *CgS* cingulate sulcus, *FEF* frontal eye field, *IP* intraparietal sulcus, *LS* lateral sulcus, *Lu* lunate sulcus, *M1* face, arm, and leg areas of the primary motor cortex, *PMd arm* arm area of the dorsal premotor area, *PMv arm* arm area of the ventral premotor area, *PrePMd* predorsal premotor area, *PreSMA* presupplementary motor area, *PS* principal sulcus, *SMA arm* arm area of the supplementary motor area, *ST* superior temporal sulcus, *TE* area of inferotemporal cortex

It is now clear that efferents from the cerebellar nuclei target multiple subdivisions of the thalamus [44] that reach widespread neocortical areas. Experiments using neurotropic viruses as transneuronal tracers have been crucial for the identification of neocortical areas that are the targets of cerebellar output (see [1] for a review). These experiments demonstrate that cerebellar output influences not only M1 but also premotor, prefrontal, and parietal areas (Fig. 1a, b). Output channels to M1 and the premotor areas are clustered in a dorsal region of the cerebellar dentate nucleus, identifying a motor domain within this nucleus. Output channels to prefrontal cortex are clustered together in a ventral (non-motor) region of the nucleus, entirely outside the motor domain [45] (Fig. 1b).

To date, all of the areas in the cerebral cortex that are targets of cerebellar output also have prominent projections to the cerebellar cortex via the pontine nuclei (Fig. 1a). Thin axon collaterals from approximately 20 % of ponto-cerebellar neurons reach the dentate nucleus [46] and may allow excitatory inputs from cortical areas, such as M1, to reach the dentate [47]. Interestingly, areas of the cerebral cortex that lack substantial projections to the cerebellum (e.g., areas 46v, 12, and TE) do not appear to be major targets of cerebellar output. If these principles apply to all cerebro-cerebellar networks, then all of the areas of cerebral cortex that project to the cerebellum may be the targets of cerebellar output. This would include such diverse areas as regions of extrastriate cortex, cingulate cortex, and the parahippocampal gyrus [48, 49].

The distinct motor and non-motor domains observed in the dentate nucleus have their counterparts in cerebellar cortex (Fig. 1c, d). The motor domain includes a region primarily in the anterior lobe (lobules III–VI) and another in the paramedian lobule and adjacent cerebellar hemisphere (HVIIB and HVIII). These regions have been shown to both receive inputs from and send outputs to M1, forming a closed-loop circuit [50] (Fig. 1c). The non-motor domain is extensive and involves the portions of the posterior vermis and hemisphere that lie between the two regions of motor representation. Regions within the non-motor domain of cerebellar cortex also participate in distinct closed-loop circuits with regions of prefrontal cortex [50] (Fig. 1d). Thus, multiple closed-loop circuits represent a fundamental feature of interactions between the cerebellum and the cerebral cortex.

The evidence that cerebellar output influences multiple areas of prefrontal and posterior parietal cortex provides the anatomical substrate for a distinct cerebellar contribution to non-motor aspects of behavior [51]. To date, most theories of cerebellar function are based on the understanding of cerebellar contributions to motor control [41, 51]. Further research is needed to determine the nature and full extent of cerebellar contributions to non-motor function.

**Fig. 2:**
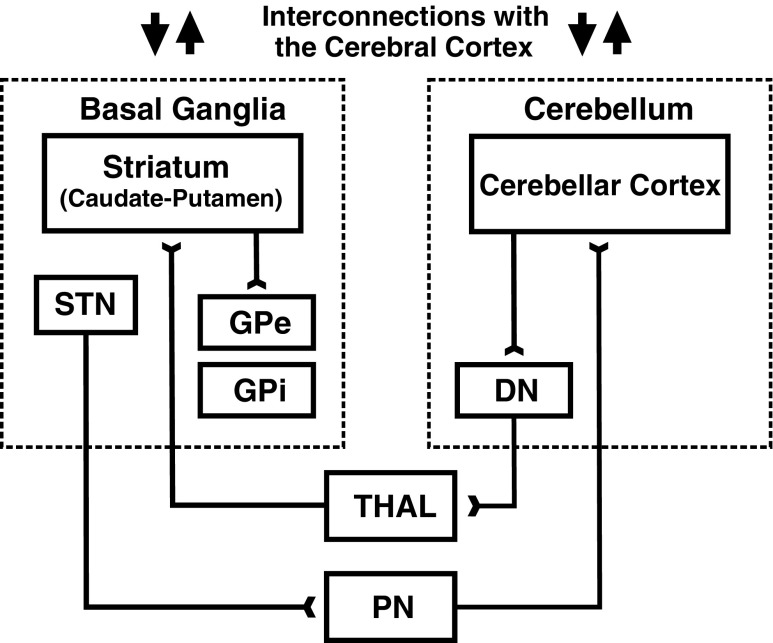
Cerebellar interconnections with the basal ganglia. Based on [12] and [13]. *DN* dentate nucleus, *GPe* external segment of the globus pallidus, *GPi* internal segment of the globus pallidus, *PN* pontine nuclei, *STN* subthalamic nucleus

The circuits that link the cerebellum with the cerebral cortex have traditionally been considered to be anatomically and functionally distinct from those that link the basal ganglia with the cerebral cortex [12, 44]. Any interactions between cerebro-cerebellar and cerebro-basal ganglia loops were thought to occur primarily at the neocortical level. Results from recent anatomical experiments challenge this perspective and provide evidence for disynaptic pathways that link the cerebellum with the basal ganglia (Fig. 2). Retrograde transneuronal transport of rabies virus demonstrated that both motor and non-motor portions of the dentate nucleus project disynaptically to the striatum (putamen and caudate) [12]. Similarly, both motor and non-motor portions of the subthalamic nucleus (STN) of the basal ganglia project disynaptically to motor and non-motor regions of cerebellar cortex [13]. The interconnections between the cerebellum and the basal ganglia provide the neural basis for cerebellar involvement in disorders typically associated with the basal ganglia (e.g., Parkinson’s disease, dystonia, Tourette syndrome and addiction), as well as in normal basal ganglia functions, such as reward-related learning [3, 37]. Furthermore, there is evidence that cerebellar output can alter striatal plasticity [52]. These new observations indicate that exploring the physiology and functions of the interconnections between the cerebellum and the basal ganglia represents an important new direction for future research.

Overall, neuroanatomical studies using virus transneuronal tracers have demonstrated that the output from the cerebellum reaches vast areas of the cerebral cortex, including regions of prefrontal and posterior parietal cortex. Furthermore, the cerebellum is reciprocally connected with the basal ganglia, indicating that the two subcortical structures are part of a densely interconnected network. These new results challenge us to discover both the entire range of behaviors that are influenced by this network and the neural computations it implements.

## Regulating the Recruitment of the Cerebellum via the Nucleo-Olivary Inhibition (P.F.M.J. Verschure, R. Zucca, I. Herreros)

The Distributed Adaptive Control theory of mind and brain describes the brain as a multi-layered control system including the body, predefined reactive control, adaptive control for state space acquisition and action shaping, and lastly the contextual control for the generation of goal-oriented plans for action [53, 54]. This raises the specific question of how these different control layers interact. The study of the cerebellum allows us to investigate in detail how reactive and adaptive control systems are interfaced. In particular, the eyeblink conditioning paradigm allows the study of the integration of cerebellar predictive commands with brainstem level reactive feedback commands. Here, we discuss this interaction between cerebellum and these lower level reflexes with the idea that it can inform hypotheses about the interaction between the cerebellum and high-level structures of the central nervous system, such as cortex and basal ganglia. In eyeblink conditioning, a subject learns to respond with an anticipatory action—the so-called conditioned response (CR)—to a naturally neutral stimulus—the conditioned stimulus (CS)—that has been coupled through the experimental training with a blink-eliciting noxious stimulus—the unconditioned stimulus (US). Experimental evidence suggests that the cerebellum provides the substrate for CR acquisition [55–63] (but see [66] and Delgado-García in [58] for alternative views). In line with that hypothesis, it has been proposed that in eyeblink conditioning, the cerebellum *substitutes* reactive reflexes by anticipatory actions [64]. However, a closer look at the resultant behavior reveals that, rather than totally replacing reactive feedback commands, feed-forward cerebellar commands are merged with them [59, 65, 68].

The difference between total or gradual replacement of reflexes by anticipatory actions becomes relevant when considering an *instrumental* version of the eyeblink conditioning paradigm where the US is provided by an air puff whose noxious effect can partially be prevented by the anticipatory blink itself [59, 61, 66, 68]. In contrast, in classical—or Pavlovian—eyeblink conditioning electrical shocks to the periorbital area are usually employed as US, in which case neither the UR nor the CR have operational value, i.e., the responses do not ameliorate the effect of the US [60, 67]. In the instrumental contingency that uses air puffs as US, the anticipatory response establishes a behavioral feedback that, as learning progresses, diminishes the sensed intensity of the aversive and learning-inducing US [65–67]. One may expect that the subject will completely avoid any aversive effect of the unconditioned stimulus by blinking preemptively, namely, to expect a complete substitution of reactive feedback by predictive feed-forward control after learning. However, behavior indicates that at learning asymptote subjects only display a partial anticipatory closure of the eyelid to the CS and will only completely close their eyes after perception of the US [59, 68–70]. This implies that a significant part of the protective action of the putative CR still takes place reactively and is thus a UR.

**Fig. 3:**
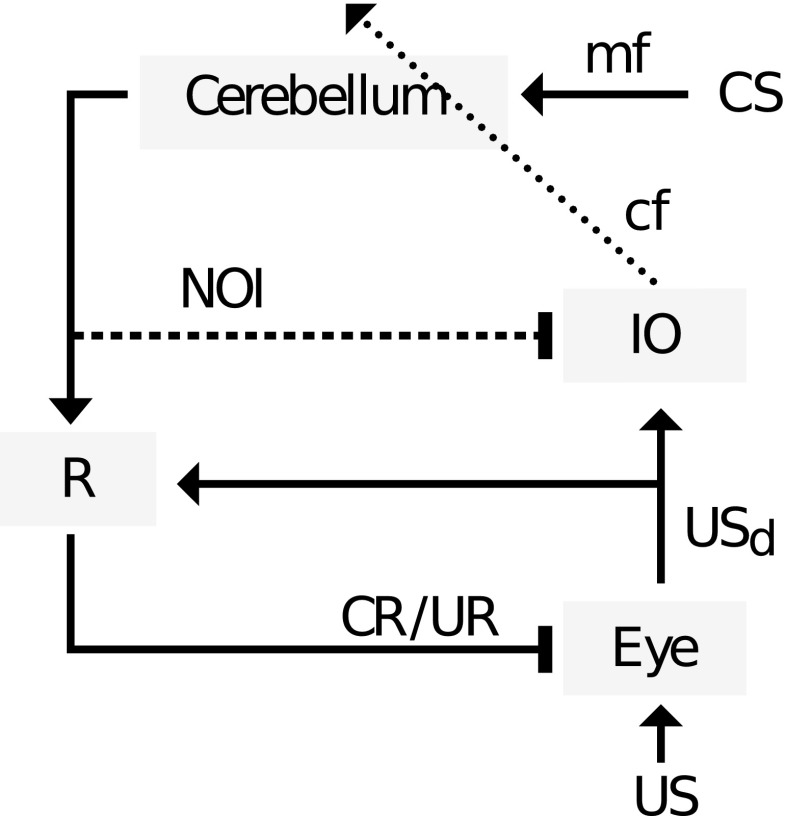
Cerebellar circuitry of the eyeblink response, with NOI. An air puff (US) is detected at the eye and elicits a neural response (USd) that by recruiting the reflex (R, oculomotor neurons) triggers the feedback reaction (UR). The effect of USd decreases proportionally to the degree of eyelid closure. The internally generated USd signal first reaches the IO and, subsequently, the cerebellum via the climbing fibers (*cf*). The convergence of the US signal with the CS information entering the cerebellum through the mossy fiber (*mf*) pathway induces plasticity in the cerebellar cortex, causing the inhibitory Purkinje cells to gradually develop a long-latency pause in their simple spikes firing. This acquired pause disinhibits target neurons in the cerebellar deep nuclei which provide the output of the circuit. This output not only reaches the downstream reflex controller (*R*), triggering an anticipatory/feed-forward response (*CR*), but also, via the NOI, counteracts the activation of the IO by the USd. In consequence, and assuming that learning stabilizes once IO activity remains at baseline, the USd signal should not be completely abolished peripherally; otherwise, the NOI would depress the IO firing below baseline introducing a negative error, which would lead to the extinction of the CR. Note that the excitatory and inhibitory outputs of the cerebellum are generated by distinct neuronal populations. *Triangular arrowheads* indicate excitatory effects and *rectangular* inhibitory ones. The CR(s)/UR(s) reaching the eye motor plant are depicted as inhibitory as they diminish the sensory consequences of the air puff US. The *dotted line* indicates the error signal, and the *dashed line* indicates a delayed connection. Note that even though we have only indicated the CS signal in the mossy fiber pathway, rich multisensory information reaches the cerebellar cortex through that pathway including, e.g., the US signal. However, by definition, only the CS will be of use in order to predict the US

Computational models of cerebellar learning coupled to a reactive controller have explained the gradual replacement of the UR by the CR in terms of internal negative feedback provided by the nucleo-olivary inhibition (NOI) [65, 71]. Via this negative feedback, the CS-dependent acquired pause of Purkinje cells firing (Purkinje cell CR) [57], leads to dis-inhibition of nucleo-olivary cells that in turn increase inhibition of the cells in the inferior olive (IO) [72] (Fig. 3). Thus, at the level of the cerebellum, this nucleo-olivary projection achieves, internally, what the behavioral negative feedback achieves externally: it reduces the intensity of the learning-inducing error signal, specifically, the driving of the activity of the IO cells above their baseline firing rate, which is in the range of 1 Hz. As a result, an air puff US can fail to initiate plasticity in the cerebellar cortex because the excitation it provides to the IO has been canceled by the NOI recruited by the cerebellar CR [73, 74]. In consequence, under the assumptions of bidirectional plasticity in the cerebellar cortex and spontaneous activity in the IO, the NOI prevents the complete substitution of a feedback by a feed-forward mode of control [65] and thus implements a mixed feedback/feed-forward controller.

We can explain this hybrid feedback/feed-forward control model from the perspective of optimality [75]. Namely, if one considers that both the protective action and the failure to avoid the US carry a cost, optimal behavior will depend on an effort/error tradeoff. In the case of eyeblink conditioning, the tradeoff may comprise of keeping the eyes open to sample visual information, versus protecting the cornea from potentially harming stimuli. NOI, by adjusting the relative weight of the internal feedback, can therefore set a balance between anticipation and reaction such that the *overall* cost is minimized [76]. In other words, cerebellar learning is not minimizing error but rather minimizing cost, including the cost of not sampling the external environment.

Mixed feed-forward and feedback control is considered the most robust control strategy, combining the efficiency of anticipatory action with the inherent robustness of feedback control. As we just observed, in nature, a balance between both modes of control can be found in the conditioning of the eye blink response and its realization in the cerebellum. Understanding this phenomenon requires complete consideration of the systems-level interactions between the cerebellar adaptive layer, the reactive brain stem motor nuclei, and the controlled plant (eyelid/nictitating membrane). Here, the use of a systems-level modelling approach has generated the testable prediction that the NOI is the critical interface regulating the coupling of cerebellum enabled predictive feed-forward control and reactive control achieved by brainstem feedback controllers. At this point, the question arises of how the NOI is itself regulated to achieve an optimal balance between the reactive and adaptive modes of control. The substantia nigra has been shown to send dopaminergic projections to the nucleo-olivary pathway [77], whose functional relevance is not yet understood. We propose that through this dopaminergic neuromodulatory output, the basal ganglia may regulate activity in the nucleo-olivary pathway and consequently control contextually the recruitment of the cerebellum. In the case of eyeblink conditioning, such control may result in a modulation of the learning rate and of the amplitude of the CR at learning asymptote. Indeed, IO cells have been shown to reproduce, in eyeblink conditioning, the encoding of temporal-difference prediction errors already found in dopaminergic cells of the basal ganglia [78], indicating an at least correlational link between dopaminergic and inferior olivary activity. In the broader context of this consensus paper, we propose that the NOI realizes a mechanism that allows to contextually adjust the contribution of distinct cortico-cerebellar loops to cortical computations and that such regulation might depend on neuromodulatory output from the basal ganglia.

The NOI pathway is a basic element of the cerebellar circuit with pervasive projections of climbing fibers throughout the cerebellar cortex. At this point, it is attractive to speculate that the possibility of regulating the degree of recruitment of the cerebellum by contextually adjusting the level of NOI would augment the computational power and versatility of brain networks that include the cerebellum, allowing them to shift contextually between more certain (prediction-based) and/or more uncertain (reactive-based) modes of control. It appears likely that this would be a more pressing control problem when we consider higher brain structures that are interfaced to the cerebellum. Indeed, given that the balancing of feedback and feed-forward control is essential for adaptive behavior, an imbalance of the two might account for pathologies such as Parkinson’s disease dyskinesia. More concretely, we can hypothesize that the over-activation of the cerebellar cortex discussed by Popa and Kishore below could result from a too strong NOI that by silencing the inferior olive increases the tonic level of activity in Purkinje cells [79]. This points to the NOI pathway as a possible target for studies with animal models of Parkinson’s disease.

## Spinocerebellar Circuitry—Consequences for the Organization of Neocortical Motor Control (H. Jörntell)

Cerebro-cerebellar communication is often implicitly assumed to refer to the cortico-pontine system and the projections back from the cerebellum to neocortex via the thalamus. However, a substantial part of the interplay between the neocortex and the cerebellum is likely to occur via the spinocerebellar systems. If the basal ganglia initiate the release of motor programs or motor command signals via the neocortex, corticospinal axons will inevitably activate large parts of the circuitry in the spinal cord. As many neuronal components in the spinal circuitry project to the cerebellum, in addition to alpha-motor neurons or spinal motor pools [87], the spinocerebellar pathways are an important source of information about the neocortical activity [80, 87].

The multitude of spinocerebellar pathways [80, 81] is of central importance to the coordination of limb movements [82]. The spinocerebellar and spino-reticulo-cerebellar pathways represent information from spinal motor circuits that are composed of various spinal interneurons. Almost all corticospinal tract axons terminate within the pool of spinal interneurons rather than the alpha-motor neurons directly [83, 84], which puts the interneurons in a key position for the majority of the motor control functions exerted by the brain. This pool of interneurons integrates the motor command signal from the neocortex with local sensory feedback, such as cutaneous information, tendon organ afferents, and muscle spindle afferents both of type I and type II. Spinal interneurons can either have direct recurrent connections that ascend all the way to the cerebellum [85] or the lateral reticular nucleus [86, 87], or they can utilize specialized ascending neurons such as the neurons of Clarke’s column [80]. These systems display differences regarding their detailed connectivity and the information they sample within the spinal cord. Consequently, the ascending projections from these systems will serve the overall function of providing the cerebellum with a wide monitoring of the activity in spinal motor circuits. Spinal interneurons are involved in the muscle synergy selection when the spinal motor circuitry is driven by descending motor control signals from the neocortex and/or subcortical motor systems [88]. Hence, the cerebellum can use the spinocerebellar systems to be informed about the relative excitatory drive on specific synergical components and on the low level motor functions resident in the spinal cord [89, 90].

In many respects, the intrinsic processing within the cerebellar cortex can to a large extent considered to be solved [91]. Mossy fiber information is transmitted through the granule cell layer and reaches the molecular layer, where the signal is given a specific synaptic weight in the Purkinje cell through learning. Due to the presence of local inhibitory interneurons, this synaptic weight can also have a negative value on the Purkinje cell. Hence, observed excitatory and inhibitory inputs to these cells in vivo can to a large extent be explained by learning [92], which is related to the climbing fiber signal the Purkinje cell receives. In light of this relatively simple circuitry function, a natural consequence is that the key function of the cerebellar cortex becomes that of a major associative element. The individual Purkinje cell has in itself massive associative power, due to the very high number of parallel fiber synapses and interneuron synapses it receives. An array of Purkinje cells that compose a functional microzone, and which targets the same group of cells in the efferent cerebellar nucleus and therefore can be considered a “super”-Purkinje cell, has a tremendous associative power [91]. One important possible consequence of this arrangement is that the cerebellum can associate many specific basal (spinal) synergy components in the right temporal order to help synthesize compound movements [89]. Although there is a debate on the pattern of convergence of mossy fiber information in individual granule cells, at least for limb controlling areas of the cerebellar cortex mossy fiber information from individual functional pathways is directly transmitted through granule cells and reflected in the output of the Purkinje cells [92, 93]. In this case, there is an unconditional transmission of information through the granule layer from individual functional pathways, which means that the information carried by these pathways can be integrated by the Purkinje cell without being dependent on concomitant input from other mossy fiber systems in contrast to what was assumed in the original Marr-Albus theory of cerebellar information processing [94].

However, there are naturally many details left to solve for the internal processing in the cerebellar cortex. One example is how the global balance of activity in the perpetually active cerebellar circuitry is maintained, where the feedback loops formed by the inhibitory Golgi cells, the nucleo-olivary inhibitory pathway, and the control of the overall Purkinje cell firing level from climbing fibers are likely to be important [95]. Another example is the role of the various neuromodulators, of which there is ample representation in the cerebellum. But with respect to the direct fast processing, which at least under limb movement control is the main functional contribution of the cerebellum, the structure and physiology of the cerebellar-cortical circuitry do not seem to provide a substrate for adding substantial computations of highly advanced functions on the information it receives. But it does allow the coupling/association of a very large amount of mossy fiber information, which in itself can represent advanced functions, as described above. In the case of spinocerebellar systems, such functions can already be represented in the information that reaches the cerebellum, and the task of the cerebellum becomes that of finding which of these many functions should be associated with each other, and under which context they should be associated.

## The DPM Architecture for Learning and Control (J. Houk)

**Fig. 4:**
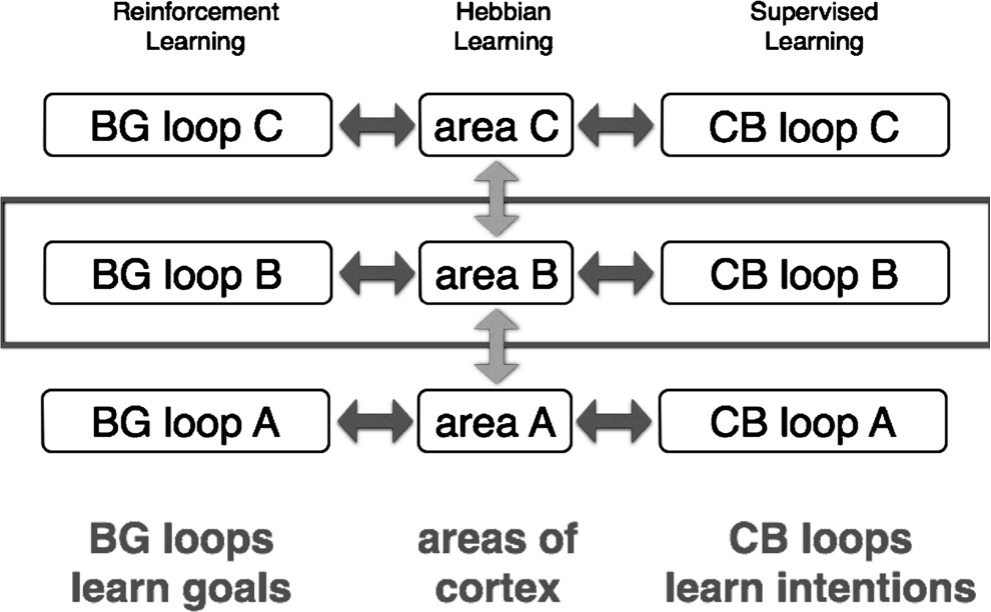
The DPM architecture for learning and control. Functionally related areas of the cerebral cortex communicate with each other via corticocortical loops [104]. In addition, most cortical areas have important learning and control loops with subcortical structures, particularly with the basal ganglia (*BG*) and with the cerebellum (*CB*). Evidence for relatively private loops through BG and through CB comes from many neuroscientific studies (e.g., [50, 96–98]). The *rectangle* outlines one distributed processing module or DPM [96]. It includes one area of cerebral cortex together with its loops of connectivity through BG and CB. The different DPMs communicate with each other mainly through reciprocal corticocortical loops

*DPMs* are distributed processing modules [96] based on the anatomy and physiology of the loops that the basal ganglia (BG) and the cerebellum (CB) form with the cerebral cortex (Ctx) (cf. [50, 97, 98]). The DPM architecture illustrated in Fig. 4 provides a powerful learning and processing system. To give a brief overview of its composite functions, BG-Ctx loops discover opportune goals through reinforcement learning [99]. CB-Ctx loops generate intentions capable of achieving these goals through supervised learning [15]. This subcortical knowledge is expressed by outputs from BG and CB that loop back to the same area of Ctx that sent the main input to the loops. These signals not only force the Ctx to perform subcortically learned actions but also promote learning in Ctx based on practice [100]. As a consequence, knowledge that is gleaned in subcortical structures is progressively exported from BG and CB to the cerebral cortex [101].

### Operations in the Subcortical Loops

**Fig. 5:**
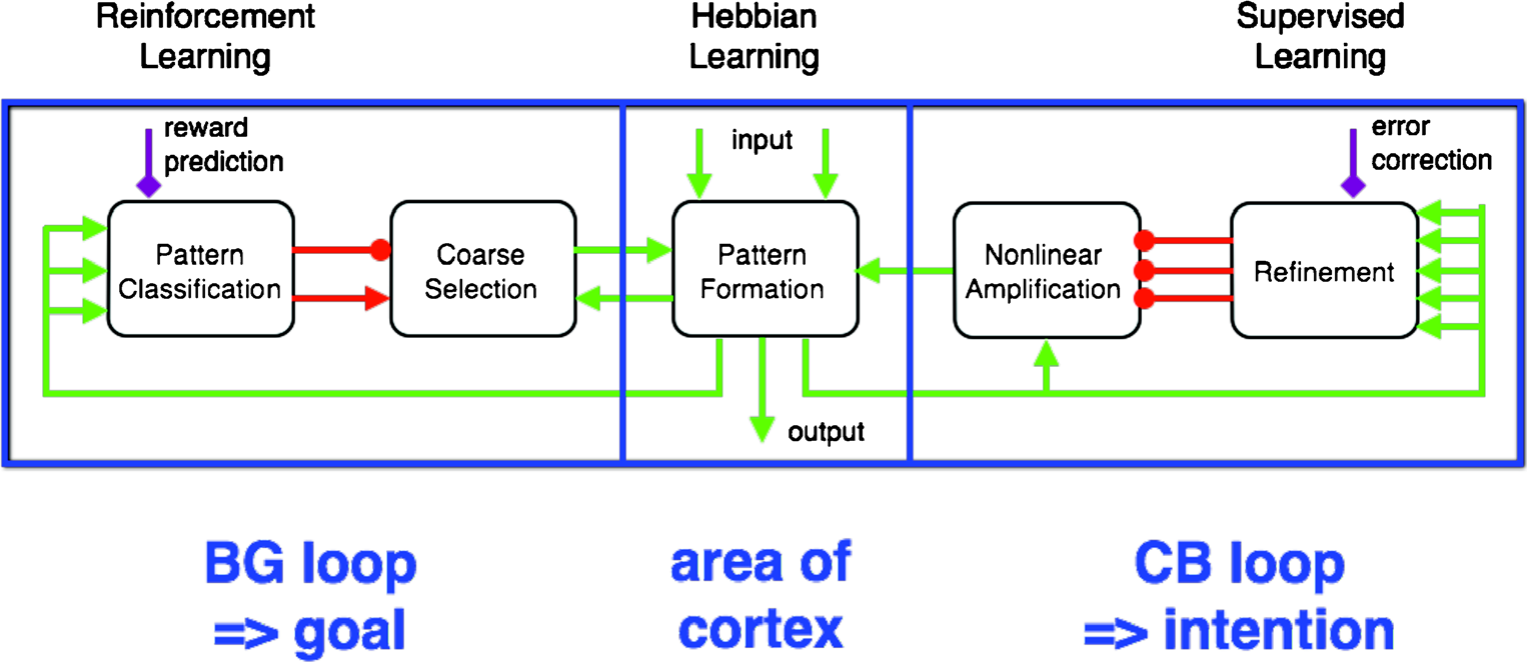
Learning and control operations in a DPM. Hebbian learning occurs in cerebral cortex. The control operation is pattern formation. Reinforcement learning occurs in basal ganglia (*BG*). The main control operation is pattern classification, which occurs in the striatum on cortical and thalamic input to spiny projection neurons (*SPNs*). Through direct (disinhibition) and indirect (inhibition of disinhibition) pathways, a coarse selection of goals is briefly stored in reciprocal corticothalamic pathways. Supervised learning occurs in the refinement stage in the cerebellar cortex, through depression of parallel fiber/Purkinje cell synapses. The positive feedback loop between the cerebellar nucleus and cerebral cortex is a working memory that is regulated by potent inhibition from Purkinje cells

Learning and control functions are defined by the operations performed in different DPM stages of any given subcortical loop (Fig. 5). Pattern formation in cerebral cortex operates much like the classical idea of an unsupervised neural network. It learns from practice (Hebbian learning) how to combine inputs in order to form useful outputs. The loops through BG and CB tell it what to practice and thus tutor the cortex to perform what has been learned in the subcortical loops. As mentioned above, the BG loop learns opportune goals, and the CB loop generates and refines intentions, such that they are capable of accomplishing the goals. On a slower time scale, the cerebral cortex learns through practice how to encode this knowledge into cortical habits. Cortical habits are stimulus–response operations that can be performed without subcortical help.

How does the BG loop learn appropriate sets of goals among which to choose? The input to BG from cortex is diverse and it converges onto a sophisticated pattern classification stage (Fig. 5), located in the striatum of the BG loop [96]. The sophisticated properties of the striatum include spiny projection neurons (SPNs) endowed with bistability and nonlinear amplification [102] and a sparse inhibitory network expressing complex dynamics [103]. Furthermore, the learning and performance of SPNs is modulated by reward prediction signals (purple diamond) sent from dopamine (DA) neurons in the midbrain. Due to this modulation, combined with a competitive process that profits from presynaptic inhibition [42], SPNs learn to detect diverse patterns of activity in cortex, patterns that encode opportune goals. In any given experience, a few winners of the competition lead to a coarse selection of preeminent goals for obtaining future reward [101].

How does the CB loop learn to generate an intention for accomplishing one of the goals that was selected by BG? Neural representations of selected goals are transmitted from cortex to two subdivisions of the cerebellum. The excitatory loop (green arrows) targets the cerebellar nucleus and engages the nonlinear amplification stage in Fig. 5. Positive feedback through cerebellar nucleus causes the loop to behave in a bistable manner [105]. Bistability is regulated by prominent inhibitory input from the cerebellar cortex (red circles), designating the refinement stage in Fig. 5. The error correction signal (purple diamond) from climbing fibers trains the principal inhibitory neurons (the Purkinje cells) to regulate bistability in a highly complex manner [106]. Transitions between a quiescent downstate and an intense upstate need to be controlled. The CB loop learns to program an intention, patiently wait for the initiation of the intention by some event, and then terminate the intention in advance of it achieving its goal. The latter is a critical step that prevents the limb from crashing into the target and amounts to predictive control as discussed by Miall in a later section. A successful intention command controls the state transitions of the loop between cerebellar nucleus and the cerebral cortex, which is a very difficult problem. This intention command serves as the ultimate output of the DPM that is transmitted from a particular area of Ctx. It may represent a movement command, a working memory, or a neural representation of a plan, depending on the particular DPM [96, 107].

### Functions Performed by Arrays of DPM

The former paragraphs explain how the three main parts of a DPM, an area of Ctx, a BG loop, and a CB loop, operate individually. Now, I would like to discuss how arrays of DPMs function in combination. The work done on mirror neurons [108] and on imitation learning [109] provides excellent topics. Mirror neurons are active when a subject performs a goal-directed action and also when the subject observes another individual performing the same action. This special correspondence between observation and action has been called *congruence*. If the DPM theory is generally applicable, it has to account for the emergence of congruence during ontogenesis. As a consequence of developmental plasticity, the infant must learn not only how to reach and grasp a specific target but also higher level actions such as bringing a morsel of food to the mouth and eating it. The infant must also learn to recognize when another individual performs the same actions. Hierarchically diverse levels of goal are learned in BG loops guided by reinforcement learning. SPNs must learn not only to fire when a subject is presented a goal for its own action but also when it observes another individual performing the same action. Meanwhile in CB loops, the infant must learn parameterizable intentions for generating outcomes capable of fulfilling the different goals. Often it is assumed that the theory of reinforcement learning specifies how basal ganglia learn primitive actions (say, muscle commands) and ensembles of them (say whole movements or longer courses of actions), but this is not biologically realistic. Muscle commands, and other kinds of intention, are clearly learned and generated in CB loops to satisfy goals that are learned and selected by BG loops. Normally, the two kinds of loop work together to select and generate appropriate actions. These learning and control operations need to be explored in simulations of DPM arrays with different parameter sets.

An early stage of imitation learning can be traced to the superior temporal sulcus (STS) using arguments that focus only on the cortical areas that are involved in the mirror neuron network [109]. Most models do not invoke the subcortical loops that learn to control each cortical node in the mirror neuron network. I agree with Caligiore and colleagues [4] that this focus of the mirror neuron field is inadequate. We now have a good understanding of how loops through BG discover opportune goals and how loops through CB adapt to generate and refine intentions that accomplish these goals. The focus of mirror neuron research needs to shift towards exploring how these goal/intention congruences are learned by arrays of DPMs. Within the hierarchy of the mirror neuron system, each DPM evokes a different meaning. In the course of developmental practice, these different kinds of knowledge are exported to the different cortical nodes for more rapid automatic execution. The latter are examples of cortical habits, which clearly may include sequential chunks of action.

What is so special about subcortical learning as opposed to learning in the cerebral cortex? Briefly stated, learning in BG and CB profits from training information as summarized in Table 1. Hebbian learning in Ctx depends only on coincidence detection with no timed-pulse training signals, just the coincidence of input and output that occurs during practice in the presence of tonic permissive factors that gate broad time intervals when learning can occur. In the BG loop, the timed training signals are brief dopamine pulses that signal predictions of when future reward may occur [99]. In the CB loop, parallel fiber input and Purkinje cell depolarization signal coincidence detection in a particular spine, and training information is conveyed by climbing fibers that detect precisely when error corrections occur [107, 110]. Thus, BG and CB receive well-timed reward and error signals as training information. Together with coincidence detection, these reward and error signals consolidate learning. When all three learning algorithms are simulated in combination, task performance can be quite remarkable [41, 111]. Testing DPM learning hypotheses is now facilitated by a new functional magnetic resonance imaging (fMRI) method for assessing the correspondence between a learning rule and the area of the brain that performs that type of learning [112].

**Table 1:** Different learning rules in cerebral cortex, basal ganglia, and cerebellum

Brain site	Cerebral cortex	Basal ganglia	Cerebellar cortex
Cellular site	Excitatory afferents onto pyramidal cells	Striatum cortical afferents onto spiny neurons	Parallel fibers onto Purkinje cells
Learning Rule & operation	Hebbian & coincidence	Reinforcement & coincidence · reward	Supervised & coincidence · error
Permissive factors	Cholinergic +	Dopaminergic +	Noradrenergic +

This table illustrates key features of three learning rules in the brain (adapted with permission from Houk [113])

## Cerebellum and Basal Ganglia Work Together for Model-Based Actions (K. Doya)

Based on the anatomical and physiological evidence available by the end of the last century, I proposed a theoretical framework that the cerebellum, the basal ganglia, and the cerebral cortex are specialized for supervised, reinforcement, and unsupervised learning, respectively [41, 111]. This view has received some support and raised some debate among theoreticians and experimentalists.

**Fig. 6:**
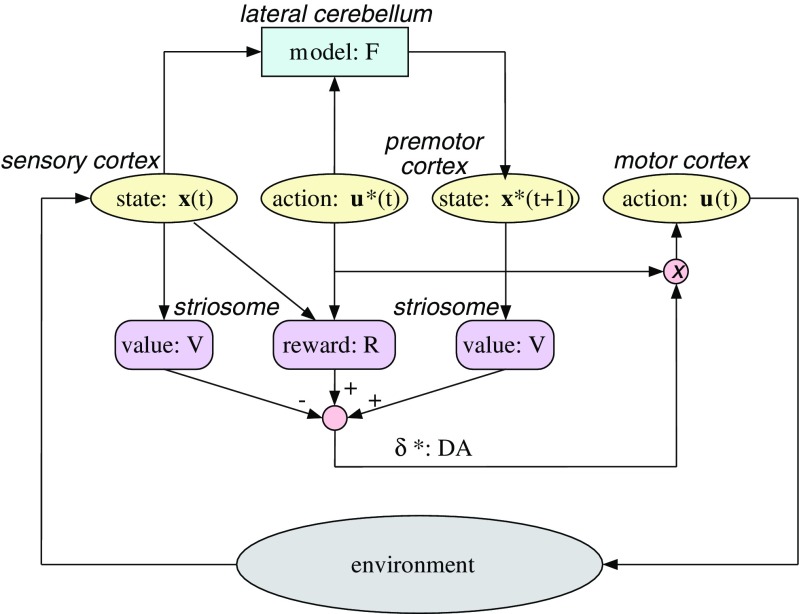
A possible implementation of model-based action selection by combining forward models in the cerebellum and reward predictors in the basal ganglia. From [111] with permission

An important combination of supervised learning and reinforcement learning is model-based action selection and learning. Supervised learning in the cerebellum, including the cerebellar cortex and nuclei, can create a “forward model” that predicts the results of executed actions [64]. Reinforcement learning in the basal ganglia can provide a “state value function” that evaluates the goodness of states. Because the dominant view at that time was that the outputs of the cerebellum and the basal ganglia form separate channels in the thalamus [6], I hypothesized that model-based action selection can be implemented by a cortico-cerebellar loop predicting the result of an imaginary action, the cortico-basal ganglia loop for evaluating its goodness, and an activation of dopamine neurons for triggering execution of the imagined action (Fig. 7 of [111], reproduced here as Fig. 6). A more specific proposal was that the striosome (or patch) compartment, which projects to dopamine neurons, learns state value function while the matrix compartment, which projects to the pallidum, learns state-action value functions [41, 99].

In the last decade, there have been significant new observations regarding the interaction between the cerebellum and the basal ganglia. The cerebellar output is disynaptically connected to the basal ganglia through dentate nucleus–centrolateral thalamus–striatum pathway [12]. It was recently demonstrated that stimulation of dentate neurons evokes short-latency (about 10 ms) responses in about half of the striatal neurons [52]. It was further revealed that the basal ganglia output is also disynaptically connected to the cerebellum through subthalamic nucleus–pontine nucleus–cerebellar cortex pathway [13]. Thus, the existence of the short-cut connections between the cerebellum and the basal ganglia is becoming a new anatomical consensus [3], but what are their computational roles?

A possible role of the dentato-thalamo-striatal pathway is model-based evaluation of action candidates. The forward model learned in the lateral cerebellum predicts the resulting sensory state for a candidate action and its output is sent through the thalamus to the striatum, which estimates the value of the predicted state. An interesting observation [52] is that optogenetic stimulation of the dentate-thalamo-striatal pathway reverts cortico-striatal long-term depression (LTD) to LTP, which had been reported to happen with dopamine stimulation [114]. Previous self-stimulation experiments showed that the striosome compartment is more effective in inducing self-stimulation than the matrix compartment [115]. It is not known whether the dentato-thalamo-striatal pathway has any preference projections, but a possible scenario is that the cerebellar input activates the striosome neurons that activate dopamine neurons.

The function of the subthalamo-ponto-cerebellar pathway is harder to interpret. The subthalamic nucleus is a part of the “indirect pathway” of the basal ganglia that is implicated in action inhibition and aversive learning [116, 117]. Thus, a possible function of the pathway is to provide a “Stop!” signal to the cerebellum for withholding ongoing movements. Another possibility, though highly speculative, is to signal “off-line” status to the cerebellum, notifying that currently the basal ganglia have withheld execution of motor programs (though inhibition of the thalamus and midbrain motor nuclei via globus pallidus and substantia nigra reticulata, respectively) so that the cerebellar internal models can be safely used for off-line mental simulation.

Here, I presented some views based on the hypothesis that the cerebellum provides internal models of the body and the environmental dynamics for model-based motor control and decisions [41, 111]. There are, however, proposals and accumulating evidence suggesting that the frontal and parietal cortices as well as the hippocampus play important roles in model-based planning and decisions [118–120]. A possible difference of the internal models provided by the cerebellum and the cortex may be that the cerebellum learns deterministic input–output mapping with its mostly feed-forward circuit, while the cortex learns joint or conditional probability models through iterative dynamics using its heavily recurrent circuit. Probabilistic models are more general, as it includes deterministic models as extreme cases, but deterministic models have the virtue of simplicity. The hippocampus may enable reuse and editing of episodic memories for planning about the future. These ideas are all speculative, but recent optogenetic tools for pathway-specific circuit manipulations can make it possible to test these hypotheses. The big challenge for us theoreticians is to provide hypotheses worthy of laborious testing.

## A Systems-Level View of Cerebellar Motor and Cognitive Function (R.C. Miall)

### A Uniform Structure and Specific Connections

The cytoarchitecture of the cerebellar cortex is strikingly consistent, both across its span and across species. This very extensive neural sheet, estimated to be equal in area of one hemisphere of the human cerebral cortex [121], is heavily interconnected with cerebral cortical areas, and recently evidence of bidirectional connections between cerebellum and basal ganglia has emerged [6, 13]. The consistent cerebellar structure and circuitry, reciprocally connected to many different extra-cerebellar structures, suggest strongly that a common neural operation is performed within each micro-complex. In contrast, cerebral cortical areas are uniquely organized, with each cortical area dedicated to a specific function or functions. Function follows form.

#### A Singular Processing Hypothesis

Three critical questions arise from this view (none of which is new and all have been raised before). The first question is what might be this proposed single operation or transformation performed by the cerebellar cortex. This is analogous to the algorithmic level of analysis that David Marr proposed 40 years ago [122]. There are still multiple alternative answers, ranging from a timing device, or associative learning network, to my preferred hypothesis, as a short-term predictor—specifically that the cerebellum contributes a state estimation towards forward modelling, predicting the causal chain from motor commands to changes in state of the modelled system [123]. We have recent evidence that the same may hold true for prediction in language processing [124].

The second question is how the cerebellar neural circuitry supports whatever singular operation it achieves (Marr’s physical level of analysis). A full answer will depend on a deeper understanding than we currently have of the processing of each cell type, the interactions between the interneurons within the cerebellum, and the consequences for information processing of the interconnections between mossy fiber inputs and Purkinje cell outputs [125]. This must include not only interactions between cells in the cerebellar cortex but also the short-range connections between cortex and deep cerebellar nuclei and between cerebellum and inferior olive. This latter connection is probably one of the most critical to understand, as the evidence for climbing fiber-driven LTD at the Purkinje cell inputs is overwhelming, and evidence for more subtle changes at other synapses within the cortical sheet is strong. So understanding what the inferior olivary teaching signal represents will determine what cortical process is sculpted and refined by these synaptic events [126].

The third question is analogous to Marr’s computational level of analysis. What is the function of the interconnected network running, for example, from parietal and sensorimotor cortical areas through the cerebellum and its output to the thalamus, and projecting back to the same sensorimotor cortical areas? Here, the answer must be given in terms of the network and cannot be answered by reference to cerebellar or cortical processes in isolation.

#### Cerebro-Cerebellar Loops

Evidence for cerebellar interactions with cortical sensorimotor areas is found at all levels—from tracer studies in non-human primates, human diffusion tensor imaging and functional connectivity measures, lesion studies, and electrical and magnetic stimulation studies. What these cannot tell us is what signals are carried or the functional role of the cerebro-cerebellar loop. Our experimental approach has been to use TMS to disrupt cerebellar operations and test consequences on behavior [124, 127]; in both studies, we showed change in behavior consistent with a loss of predictions generated by the cerebellum—the former study tested linguistic prediction, the latter reaching behavior. We are using functional imaging to understand the co-activation of cerebral and cerebellar areas [129] and more recently to explore how their connectivity changes as a consequence of learning [130]. We have also used tDCS to manipulate either cerebellar or cerebral excitability, and this is highlighting a secondary modulatory level of cerebellar-cerebral interaction [131–133]. In detail, we have shown corresponding changes in cognitive tasks when we depress cerebellar activity with cathodal tDCS or activate frontal cortex with anodal tDCS. We believe the former effect is mediated by cerebellar disinhibition of cerebral cortex; both Lago-Rodriguez and Galea and Popa and Kishore discuss this mechanism in more detail below.

**Fig. 7:**
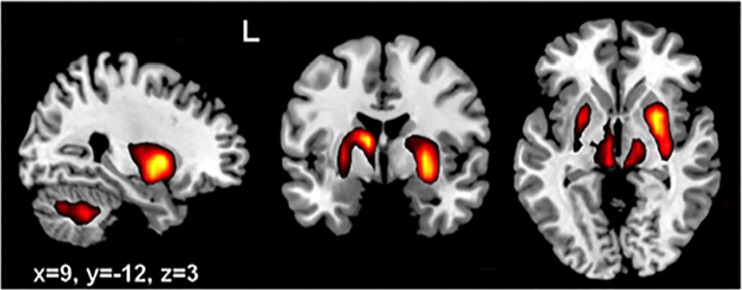
Functional connectivity within a network encompassing cerebellar nuclei, the thalamus, and basal ganglia was significantly greater than at baseline 30 min after exposure to an implicit sequence learning task. Similar connectivity increase was seen for explicit learning; in both cases, this declined after 6 h. From [155] with permission

However, two results are of particular mention. We first reported that the functional connectivity within the cerebellum and a separate fronto-parietal network was enhanced shortly after a period of sensory motor learning [130]. The implication is that cerebellar processes may contribute to the consolidation of a motor skill, probably leading to long-term change in both cerebellar and cerebral sites. Second, after a similar period of learning in the serial reaction time task (SRTT, a sequence learning paradigm) [155], we again saw short-term changes within 10 min in cerebellar, frontal, and occipital areas that led to later changes, measured at 6 h after learning, within sensorimotor cortex and medio-temporal areas, perhaps reflecting long-term memory store. One group, learning under explicit conditions, showed early increased connectivity within cerebello-frontal and visual cortical areas and later increased connectivity in sensorimotor cortex. The other group learning under implicit conditions showed only the sensorimotor cortical network early after learning, shifting to a medial temporal network after consolidation. Interestingly in the intermediate period, 30 min after learning, a network involving cerebellar nuclei, thalamus, and basal ganglia was enhanced in both groups (Fig. 7) and may reflect the transition between early cerebellar events and later cerebral cortical consolidation. This pattern suggests that learning in the SRTT involves both cerebellar change but also reorganization of information held by frontal and sensorimotor cortical areas. This is potentially consistent with Houk’s DPM architecture outlined above, in which he suggests ‘”knowledge that is gleaned in subcortical structures is progressively exported from BG and CB to the cerebral cortex [101].” However, Doya’s computational account of the dentato-thalamo-striatal pathway may provide a stronger hypothesis. The cerebellum may predict the sensory state of an action and feed it to the basal ganglia, which would estimate the behavioral value of the new state. What subthalamo-ponto-cerebellar connections might convey is less obvious; Doya speculates above they may provide a stop signal to block motor output or a switch to tell the cerebellum its predictions are “off-line” and can be used for exploratory simulation. One further possibility, driven by evidence that reinforcers differentially influence motor learning [128], is that the basal ganglia might modulate the cerebellum’s sensitivity to errors, priming the cerebellum to weight its predictions or to update its forward models based on predicted reward. These bilateral connections might thus be exposed by enhanced dentato-thalamo-ganglionic connectivity during the consolidation of the new skill. It would imply, in both Houk’s and Doya’s accounts, that the thalamus is a nodal point in the network linking cerebral areas, basal ganglia, and cerebellum, as shown in Fig. 7.

To conclude, neuroanatomy suggests a singular role for the cerebellum, within multiple functionally distinct cerebro-cerebello-thalamic circuits. Evidence is accumulating that this role is in prediction and in learning through experience. Medium-term, post-learning, changes in connectivity between the cerebellum and the cerebral cortex reflect the consolidation of these processes and may depend upon cerebellar and basal ganglia interactions.

## What Have We Learnt from Non-Invasive Brain Stimulation Studies Regarding the Role of the Cerebellum and Its Interactions with Other Brain Regions in Motor Control and Learning? (A. Lago-Rodriguez, J.M. Galea)

Over several decades, a substantial body of work has investigated the role of the cerebellum and its connections with other brain areas in human motor control and learning [34]. Non-invasive brain stimulation techniques such as TMS and tDCS have been used to probe the functional importance of cerebellar connectivity in motor control/learning. In general, tDCS has been used to locally modulate cerebellar excitability [156], whereas TMS has been applied to either locally disrupt cerebellar processes [127] or to evaluate cerebellar-primary motor cortex (M1) inhibitory connectivity (cerebellar brain inhibition (CBI)) [157]. CBI takes advantage of the cerebellum’s inhibitory connections with M1, applying a conditioning pulse of TMS to the cerebellum prior to stimulating the M1. The following section will provide an overview of what we have learnt using non-invasive brain stimulation regarding the role of the cerebellum and its connections with other brain regions in motor control/learning. We will focus on studies of upper limb reaching movements and highlight the issues still to be resolved.

### Motor Control

The cerebellum is essential for accurate reaching performance. For example, patients with cerebellar lesions often show highly variable and imprecise reaching behavior [158]. TMS evidence from both healthy participants and cerebellar ataxic patients [159] suggest that human’s ability to perform accurate reaching is highly dependent on the strength of connectivity between the cerebellum and M1. Put simply, increased inhibitory output from the cerebellum to M1 (CBI) correlates with movement precision, where stronger inhibition is associated with greater precision [157]. Interestingly, participant’s dominant hand showed greater CBI and reaching precision than their non-dominant hand. It is tempting to suggest that stronger inhibition by the cerebellum may underlie the preference for dominant hand movements. However, it is important to point out that the observed correlations between CBI and behavioral precision do not speak to causality. Therefore, it is also possible that hand preference leads to a practice effect which enhance cerebellar connectivity [157]. What role could the connectivity between the cerebellum and M1 play in reaching accuracy? Accurate state estimation (e.g., estimating current limb position) is vital for the precise control of reaching movements. It is believed that a cerebellar forward model, which predicts the sensory consequences of motor commands, is crucial for accurate and up-to-date state estimation [127]. The correlation between behavioral precision and higher CBI would be consistent with this framework, particularly if one makes the assertion that increased CBI is indicative of a stronger forward model [157].

### Motor Adaptation

In addition to motor control, the cerebellum plays a pivotal role in error-based motor learning, often referred to as motor adaptation [31]. This process is crucial for maintaining accurate reaching behavior in response to novel environments that cause errors in performance. Modulation of cerebellar activity by tDCS leads to polarity-dependent changes in motor adaptation rates to visuomotor [31], locomotor [160], and dynamic [161] novel perturbations. Whereas anodal tDCS leads to faster motor adaptation, cathodal stimulation decreases adaptation rates [31, 160, 161]. This clearly identifies a prominent role of the cerebellum in motor adaptation; however, how important are its connections to other brain areas? Previous work has shown that the cerebellum decreases its inhibition over M1 (decreased CBI) when movement corrections (motor adaptation) are required to respond to environmental changes [162, 163]. These results are in line with a reduction of cerebellar Purkinje cells activity in response to error signals and support the notion that mechanisms similar to LTD are the most important neurophysiological processes underlying the cerebellar contribution to motor adaptation [164].

### Outstanding Issues

#### Posterior Parietal Cortex (PPC)

Current theories of motor control suggest that the anatomical connection between the cerebellum and PPC plays a critical role in maintaining accurate voluntary movements [34]. Despite this, no brain stimulation work has investigated the functional importance of cerebellar-PPC connectivity to motor control and adaptation. One reason for this is the complexity involved in such a study. For example, this issue could be investigated by comparing the influence of concurrent cerebellar tDCS on cerebellar-M1 and PPC-M1 paired-pule TMS. This complex brain stimulation design would then have to be coupled with a motor task.

#### Basal Ganglia/Frontal Cortex

There is now increasing evidence that error-based motor learning occurs through multiple processes that are not all cerebellar-dependent (reinforcement learning/use-dependent learning/explicit strategies) [165]. Although discussion of these processes is beyond this current review, it is important to mention that they can often take place concurrently or even in a competing manner [166]. However, very little is known regarding the neural basis of the interaction between cerebellar-dependent motor adaptation and possible frontal/basal ganglia-dependent learning (explicit strategies/reinforcement learning). Although we know that the cerebellum has connections to both the basal ganglia and the frontal cortex [37], the functional relevance of these connections to the interaction between different learning processes is currently unknown. Once again, the complexity involved in studying these connections with brain stimulation is significant. One possible solution would be to examine the interaction of these motor learning processes during concurrent brain stimulation (TMS/tDCS) and fMRI.

## Cerebellar Modulation of Cortical Plasticity in Basal Ganglia-Related Movement Disorders (T. Popa, A. Kishore)

**Fig. 8:**
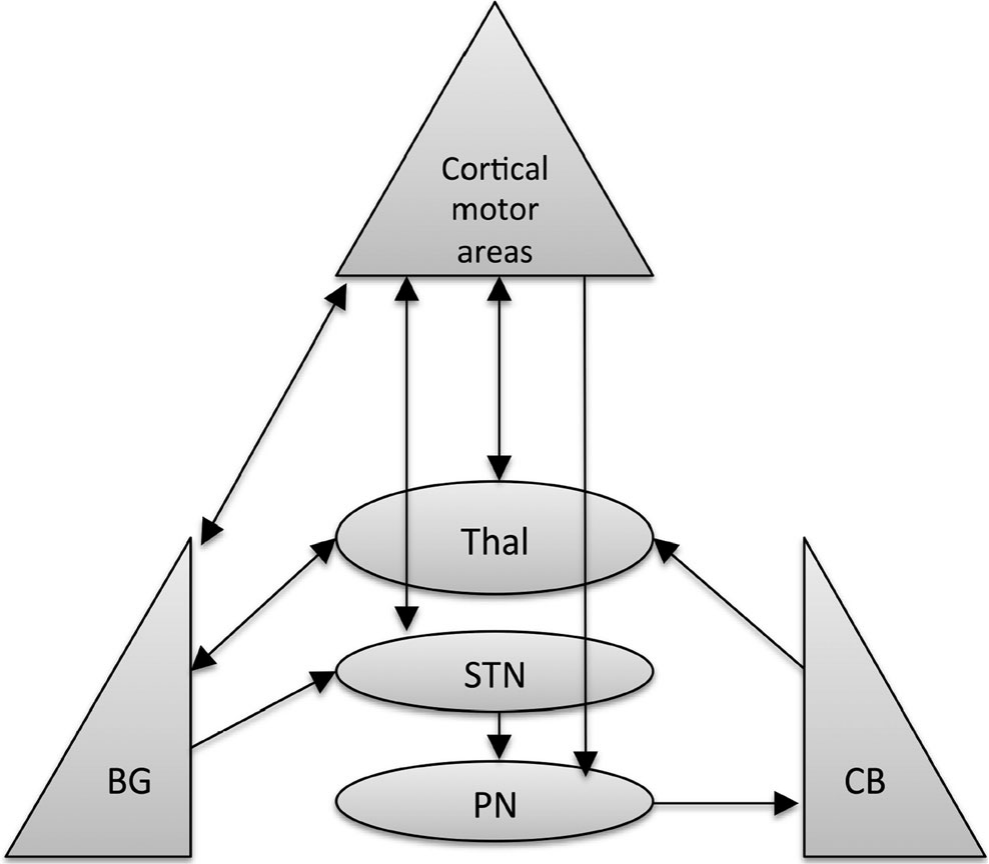
Schematic representation of the known main communication pathways among the equally important three nodes of the motor control network

Cerebellum, basal ganglia, and motor areas in the frontal cortex are the major nodes in the motor network, and they have unique cytoarchitectural properties, reciprocal connectivity, and synaptic plasticity mechanisms that allow efficient communication among them (Fig. 8). The discovery of reciprocal, topography-specific, subcortical connections between cerebellum and basal ganglia in primates [12] indicates that the basal ganglia and cerebellar motor networks are not independent information processing systems but can exchange information between them in real-time to provide appropriate inputs to the frontal cortices. In rats, under physiological conditions, the cerebellum modulates cortico-striatal plasticity through these connections [52], and the same pathway can also transmit aberrant cerebellar activity to basal ganglia, generating dystonia and dyskinesias in animals. Cerebellum also modulates cortical sensorimotor integration [167, 168] and is important for acquiring and maintaining [169] new cortico-spinal integrations. In young healthy humans, non-invasive stimulation studies demonstrated that cerebellum exerts a bidirectional modulation only on heterosynaptic motor cortex (M1) plasticity [170], which is dependent on peripheral sensory information reaching the M1 through complex polysynaptic pathways [171]. The cortical heterosynaptic plasticity can be suppressed by excitation of the cerebellar cortex or amplified, prolonged, and rendered less topography-specific by inhibition of the cerebellar cortex [170]. These findings could be an indirect evidence of the fine-tuning mechanism in place in the cerebello-cortical network to rapidly adapt the motor commands to new environmental challenges during movement planning. If so, excitation of the cerebellar cortex would inhibit the ipsilateral dentate output and thereby the sensory relay to M1, thus leaving M1 less receptive to any new motor programs. In contrast, the inhibition of cerebellar cortex would disinhibit the dentate output, which would facilitate the sensory relay to M1, permitting the selection of new motor programs [170]. From this perspective, such preparations of continuously anticipating and pre-planning motor programs for simple movements through cerebellar modulation would assist the efficient execution of complex movements. Pathologies, in which cerebellar bidirectional adjustment of M1 plasticity is impaired, can therefore be expected to affect motor performance and motor learning that need fine online adjustments.

The abnormalities in M1 plasticity in patients with disorders such as primary dystonia and Parkinson’s disease (PD) are generally attributed to abnormal basal ganglia input to the M1. Non-invasive studies testing the efficiency of cerebellar modulation of cortical excitability were recently reported in patients with writer’s cramp [172, 173]. Though the ongoing M1 plasticity was not different from age-matched healthy volunteers, both excitation and inhibition of the cerebellar cortex were ineffective in modulating the heterosynaptic cortical plasticity in patients with writer’s cramp. Also, the extent of modulation of motor cortex plasticity by cerebellar inhibition had an impact on the efficiency of online adaptation in a sensorimotor adaptation task [172]. This deficiency might lead to degradation, with time, of the specific, vulnerable, motor programs for tasks that require reinforcement by online sensorimotor adaptation such as writing. However, clinically relevant improvement in dystonia was not observed following a single session of artificial cerebellar excitation [173] or inhibition in patients with writer’s cramp [174]. This is not surprising since abnormal motor programs for writing must have been reinforced over years, before becoming manifest and it might require many cerebellar stimulation sessions to reverse this process [175].

Studies of plasticity changes in M1 in early PD report variable results. The results are more consistent regarding the loss of bidirectional plasticity of M1 in advanced PD patients with motor complications of levodopa treatment [176–178]. There is indirect evidence that cerebellar modulation of M1 plasticity is impaired in advanced PD patients with levodopa-induced dyskinesias. Repeated sessions of cerebellar inhibition could enhance the M1 heterosynaptic plasticity [179] and concurrently reduce the severity of dyskinesias [179, 180]: the greater the enhancement of M1 plasticity following cerebellar inhibition, the greater was dyskinesias reduction. This raises an interesting question whether there is a state of deficient cerebellar inhibitory modulation or excessive cerebellar excitation in advanced PD.

**Fig. 9:**
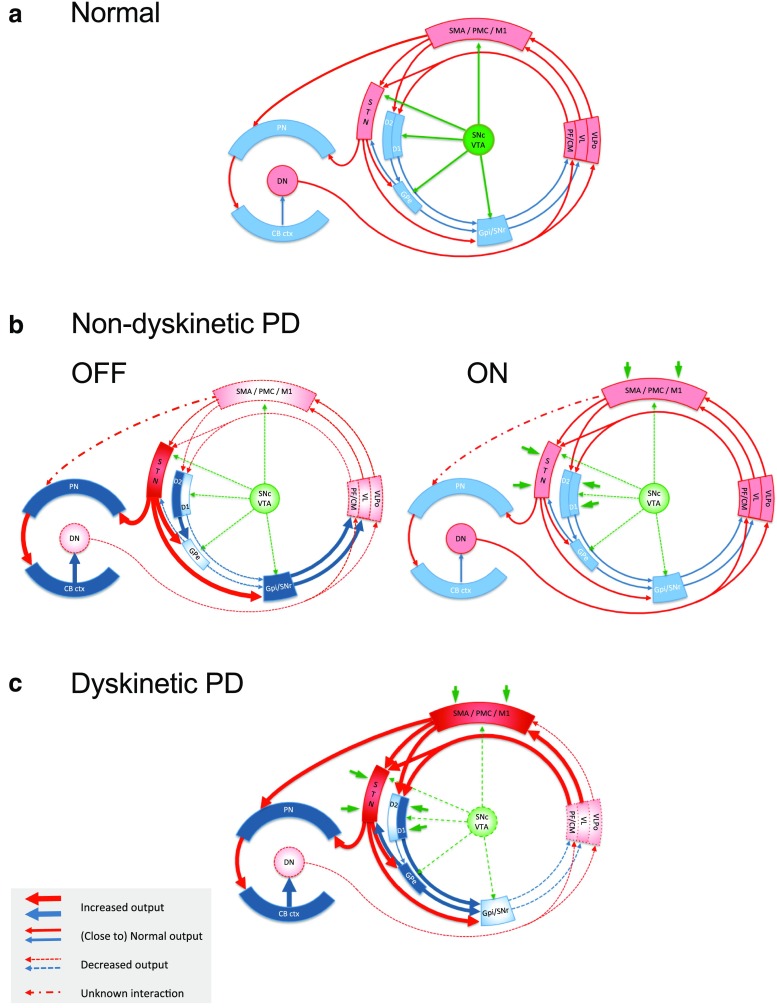
Schematic representation of the basal ganglia-thalamo-cortical loop, the cerebello-thalamo-cortical loop, and the interaction between the two in health (**a**), in non-dyskinetic Parkinson’s disease, after levodopa withdrawal (OFF) and after regular dose of levodopa (ON) (**b**), and in advanced Parkinson’s disease with levodopa-induced dyskinesia (**c**). *Red arrows* represent glutamatergic projections; *blue arrows* represent GABA-ergic projections; *green arrows* represent dopaminergic projections; *dark green arrows* in panels **b** and **c** represent the exogenous dopamine from levodopa. The *shades* of the blocks represent the activity of the respective network nodes. The STN is overactive because of cortical glutamatergic over activity during dyskinesias and from loss of GPe inhibition in OFF. The STN over activity locks cerebellar cortex in a persistent hyperactive state and interferes with its sensory processing function. The behavior of the cortico-ponto-cerebellar projections in non-dyskinetic PD in ON is not reported so far and is predicted by this model to be close to normal (*CB ctx* cerebellar cortex, *CM* centromedian thalamic nucleus, *D1/D2* dopamine receptor types of the striatal medium spiny neurons, *DN* dentate nucleus, *GPe* globus pallidus externus, *GPi* globus pallidus internus, *M1* primary motor cortex, *PF* parafascicular nucleus, *PMC* premotor cortex, *PN* pontine nuclei, *SMA* supplementary motor area, *SNc* substantia pars compacta, *SNr* substantia pars reticulata, *STN* subthalamic nucleus, *VL* ventrolateral thalamic nucleus, *VLPo* ventro-latero-posterior thalamic nucleus pars oralis, *VTA* ventral tegmental area)

Whether altered balance between the activity in the basal ganglia and cerebellum in advanced Parkinson’s disease is caused by the primary pathology (i.e., dopamine depletion in the basal ganglia) or by a maladaptive compensation (e.g., increased activity in the cerebellum) remains an important point to be clarified in the future, which might lead to new therapeutic approaches, including new surgical targets. At this moment, both hypotheses are equally plausible and not mutually exclusive: such a state of imbalance in the cerebellum could affect basal ganglia functions and generate dyskinesias just as in the rat model [167], or the aberrant basal ganglia activity in advanced PD could affect the bidirectional cerebellar modulation of M1 plasticity [179]. Based on the indirect evidence of cerebellar involvement in advanced PD and the state of cerebellar hyperexcitation in the levodopa-naïve, MPTP-treated primates [181], a model was recently proposed [182] that considers cerebellar, basal ganglia, and motor cortical networks as interlinked and their abnormal interactions as central to the generation of abnormal movements in both parkinsonism and levodopa-induced dyskinesias (Fig. 9). This model needs further validation in animal models of PD and levodopa-induced dyskinesias.

Another important detail concerning this heavily interdependent circuit is the increased activity in the cerebellar cortex that inhibits the cerebellar output from the nuclei, which, in turn, project not only to the thalamic relays but also to key hubs in the brainstem, like the inferior olive. However, most of the spontaneous activity in the cerebellar cortex is driven by intrinsic (non-synaptic) mechanisms and the level of spontaneous activity in Purkinje cells is reciprocally regulated by the activity of the inferior olive (through the nucleo-olivary inhibition). Any regulation of the strength of this negative link remotely from other brain structures or artificially (pharmacologically or with invasive-non-invasive stimulation) could be a means to adjust the level of spontaneous activity in the cerebellar cortex and subsequently in the whole network.

## The Basal Ganglia-Cortical-Cerebellar Network in Parkinson’s Resting Tremor (D. Caligiore, R.C. Helmich, M. Dirkx, G. Baldassarre)

**Fig. 10:**
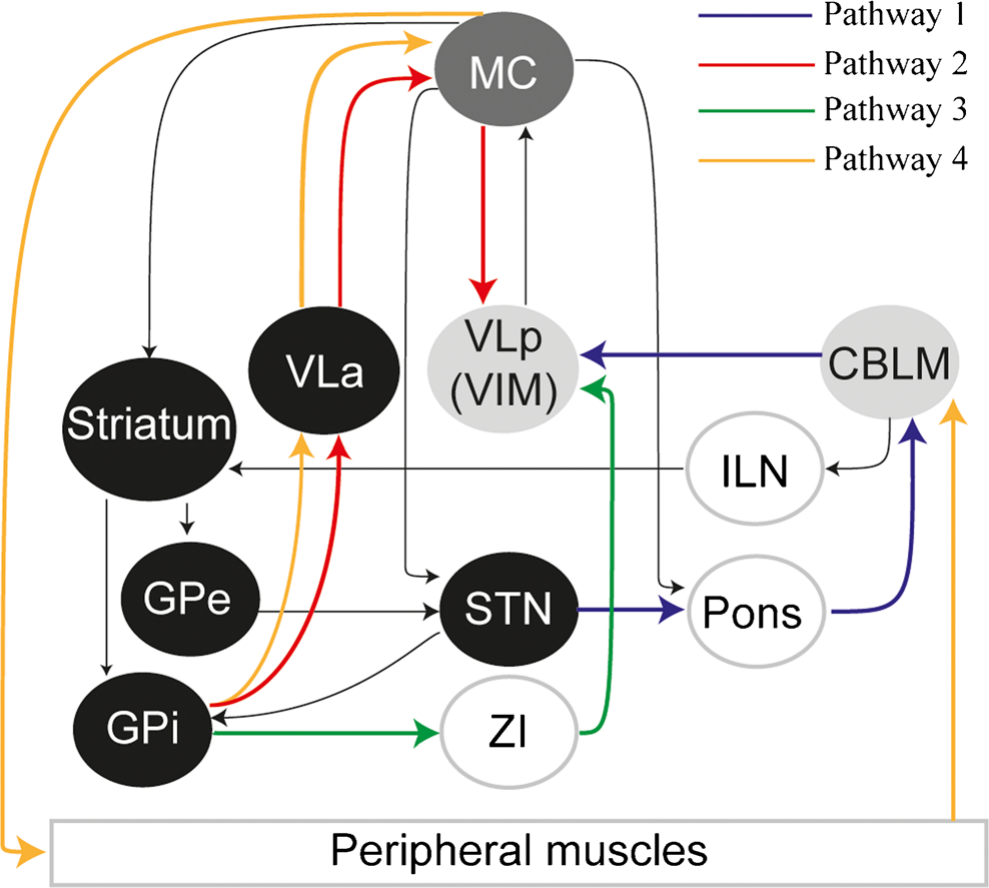
Figure showing the basal ganglia (in *black*) and the cerebello-thalamic pathway (in *light gray*) including the motor cortex (in *dark gray*) where both circuits anatomically converge. Both the basal ganglia and the cerebello-thalamic loop are involved in the production of Parkinson’s resting tremor, but it is unclear how they interact. Here we show four possible anatomical pathways through which the basal ganglia may influence the cerebello-thalamic circuit (*colored lines*). Pathway 1 connects both circuits through the pons (*blue*), pathway 2 through the motor cortex (*red*), pathway 3 through the zona incerta (*green*), and pathway 4 through the peripheral muscles (*orange*). *CBLM* cerebellum, *GPi* globus pallidus internus, *GPe* globus pallidus externus, *ILN* interlaminar nuclei, *MC* motor cortex, *STN* subthalamic nucleus, *VLa* anterior part of venterolateral nucleus of thalamus, *VLp* posterior part of venterolateral nucleus of thalamus, *VIM* ventral intermediate nucleus of thalamus, *ZI* zona incerta

It is commonly acknowledged that resting tremor in PD has a central origin, but the localization of the central oscillator (or oscillators) is still debated. There is converging evidence from metabolic imaging, electrophysiology, and deep brain stimulation studies that both the basal ganglia and the cerebello-thalamo-cortical circuit are causally involved in PD resting tremor [36]. Recently, Helmich and colleagues have proposed the “dimmer-switch” hypothesis to explain the different roles played by these two circuits in PD resting tremor [36, 183]. According to this *systems-level* perspective, the basal ganglia may work as a light switch triggering tremor-related responses in the cerebello-thalamo-cortical circuit that, in turn, generates the tremor modulating its amplitude like a light dimmer. This view is in line with previous theoretical proposals highlighting the roles of basal ganglia for triggering movement initiation and of cerebellum for movement amplification of motor patterns [42]. These physiological roles arise from the unique architecture of each circuit. Specifically, the balance between activity in the direct and indirect pathways in the basal ganglia enables the GPi to select one action while inhibiting others [184, 185]. Furthermore, the strong rhythmic (6–10 Hz) properties of the cerebello-thalamo-cortical circuit—which have been related to motor timing [186, 187]—may enable this circuit to estimate limb position in relation to the movement goal [41]. This suggests that existing functional modules—involved in voluntary action selection in healthy subjects—are driven into producing involuntary actions (resting tremor) in Parkinson’s disease, but it is not clear why this happens. Functional changes in the neurotransmitter systems projecting to these circuits may play a role: alterations in the dopaminergic, serotonergic, and noradrenergic systems have all been associated with PD resting tremor [183, 188, 189]. The dimmer-switch hypothesis also does not specify “how” the basal ganglia interact with the cerebello-thalamo-cortical network. Here we discuss four possible pathways that may play a role in this (Fig. 10), and we discuss research strategies to distinguish between these possibilities. Finally, we highlight how a systems-level view on PD may lead to the development of new treatments.

Studies in non-human primates have shown the presence of several anatomical pathways between the basal ganglia and the cerebello-thalamo-cortical circuit. First, the STN has both afferent and efferent connections with the cerebello-thalamo-cortical circuit: it receives direct anatomical projections from the primary motor cortex and sends disynaptic projections to the cerebellar cortex through the pontine nuclei [4, 13]. In PD, the activity of excitatory/glutamatergic output neurons of the STN is increased [190], and this may trigger the cerebellar cortex via the pontine nuclei, as these projections are largely glutamergic [13]. In this way, the STN may work as a switch that triggers, through the subthalamo-ponto-cerebellar pathway, abnormal activity in the cerebello-thalamo-cortical circuit. Although this hypothesis explains why DBS of the STN and ventral intermediate nucleus (VIM) are very effective in treating resting tremor [191, 192], it diminishes the role of the internal globus pallidus (GPi) in the generation of tremor. This does not explain the presence of tremor-related activity in the GPi [183, 193] and the fact that DBS targeting the GPi effectively reduces tremor [194]. Second, the basal ganglia and the cerebello-thalamo-cortical circuit may interact at the level of the motor cortex, where both circuits anatomically converge [195]. Specifically, the GPi sends GABA-ergic projections to the anterior part of the ventrolateral thalamus (VLa) which in turn sends excitatory efferents to the motor cortex that projects to the posterior part of the ventrolateral thalamus (VLp, also termed VIM), which receives cerebellar projections [196]. This hypothesis would explain why DBS of the STN, GPi, and VIM are all effective in treating tremor [191, 192, 194]. Third, the basal ganglia may engage the cerebello-thalamo-cortical circuit also through the zona incerta, which receives projections from the GPi and sends GABA-ergic projections to the VLp [197]. Finally, the basal ganglia may interact with the cerebello-thalamo-cortical circuit through the peripheral nervous system. That is, after tremor initiation by the basal ganglia, tremor-related somatosensory afferents transmitted to the cerebellum may engage the cerebello-thalamo-cortical circuit [198]. However, this hypothesis does not fit with data showing that peripheral deafferentation, peripheral anesthesia of tremulous muscles, and mechanical perturbations have little effect on PD tremor (for a review, see [36]). To conclude, there is currently no definite evidence which of the pathways connecting the basal ganglia with the cerebello-thalamo-cortical circuit contribute to PD tremor.

Given the complex involvement of different neural circuits, a systems-level perspective on PD tremor is required to answer this question. Systems-level questions require systems-level outcome measures, and the development of new analysis tools has made this possible. For example, rather than testing for altered activity in a set of brain regions, or altered functional connectivity between two different brain regions, dynamic causal modelling (DCM) allows one to statistically compare different network configurations based on fMRI data. A second approach could rely on computational modelling [39, 199] that has the advantage to allow one to directly measure the effects of adding or removing specific pathways on the oscillatory output of the system. Finally, animal models of tremulous PD may be helpful to test whether the resection of specific pathways interferes with tremor. For example, this approach has been applied to a mouse model of dystonia where resection of the anatomical pathway between cerebellum and basal ganglia was shown to reduce dystonic symptoms [200].

A systems-level view of tremor may improve existing treatments, for example by tailoring GPi-DBS to ongoing activity in the motor cortex [201]. It may also facilitate the development of new treatments. For example, computational models informed by functional imaging may allow us to test new anti-tremor treatments “in silico” before transferring them to animals or patients [199].

## Summary and Conclusions

The nine manuscripts collected in this consensus paper involved 18 scholars whose expertise covers different scientific disciplines including, among others, neurophysiology, theoretical neuroscience, and neuropsychology. A first notable fact that emerges from the paper is that, although starting from such different backgrounds, all the contributors agreed on the importance of studying the cerebellar function from an integrative perspective that takes into account the interplay between cerebellar, cortical, and basal ganglia networks. Bostan and Strick underline how the reciprocal anatomical loops between the cerebellum and the cerebral cortex involve both motor and non-motor cortical areas. Verschure et al. further support and expand the analysis of Bostan and Strick by underlining the role of the nucleo-olivary pathway to manage the contribution of feedback and feed-forward aspects of motor control. Similarly, Jörntell underlines how the spinal inputs into the cerebellum allow it to be informed about the excitatory drive on low level motor functions, so also adding the spinal cord to the systems-level view on cerebellum. Furthermore, Bostan and Strick emphasize their recent findings on the connectivity between the cerebellum and the basal ganglia. While most of the authors propose possible systems-level effects of these bidirectional cerebellar-basal ganglia anatomical pathways, Houk decided to not explicitly include them in his entry. Houk claims that they are too sparse and there are not enough data to properly address the question about which role they might play in the cerebello-cortical-basal ganglia system. Houk only proposes here that such collateral synapses might grow in strength the gain of the positive feedback managed by the cerebellar-cortical loops discussed in his entry and, as a consequence, such loops would become extremely difficult to control. Aside from this, there is unanimous agreement between all the authors of the consensus paper that future experimental and computational work is needed to understand the function of cerebellar-basal ganglia circuitry in both motor and non-motor functions. In this respect, Doya proposes novel untested theories regarding the role of such cerebellar-basal ganglia loop.

The cross-disciplinary discussion between the contributors allows the identification of some points of agreement and disagreement about *specific mechanisms* through which the cerebellum expresses its functions in synergy with cortex and basal ganglia according to a systems-level view. Below, we summarize the result of such discussion by considering three main topics addressed by the authors: (i) cerebellar systems-level mechanisms for learning and adaptation; (ii) cerebellar systems-level mechanisms underlying dystonia and Parkinson’s disease; and (iii) techniques for systems-level analysis of cerebellar functions.

### Cerebellar Systems-Level Mechanisms for Learning and Adaptation

Houk reviews his previous proposal claiming that cerebellum, cortex, and basal ganglia form DPMs where basal ganglia-thalamo-cortical loops discover goals through reinforcement learning processes; cerebello-thalamo-cortical loops learn to generate intentions through supervised learning. According to this proposal, knowledge that is gleaned in basal ganglia and cerebellum is progressively transferred to the cerebral cortex. The basal ganglia-cortex and cerebellum-cortex loops tutor the cortex to learn what has been learned in the subcortical loops. Verschure et al. agree with this view and underline how the learning processes that Houk proposes for cerebellum, basal ganglia, and cortex are compatible with Doya’s attribution of supervised, unsupervised, and reward-based learning to, respectively, cerebellum, cortex, and basal ganglia. Interestingly, Verschure et al. also suggest that the regulation of the cerebellar feedback inhibition to the olive discussed in their entry may be the mechanism by which the cerebellar contribution to each DPM gradually adjusts to contextual requirements. Indeed, the DPM systems-level architecture may benefit of a mechanism allowing a flexible reconfiguration (or weighting) of its basic layout. The network could entertain modes of behavior that are more “cerebello-centric” (for instance to adapt to a new change in a sensorimotor contingency) to “cortex-centric” behaviors. Similarly, Miall agrees with the general ideas proposed by Houk, even if he mainly stresses the role of the cerebellum as a predictor.

Doya elaborates his previous theory of motor control and learning according to which the cerebellum, the basal ganglia, and the cerebral cortex are specialized for supervised, reinforcement, and unsupervised learning, respectively, in order to account for the recent findings on the circuitry between the cerebellum and the basal ganglia reviewed by Bostan and Strick. In particular, the author suggests that a possible role of the dentato-thalamo-striatal pathway may be a model-based evaluation of action candidates during the reinforcement learning processes. On the other side, the function of the subthalamo-ponto-cerebellar pathway may be to provide a “Stop!” signal to the cerebellum to activate a motor program for withholding ongoing movements. Another possibility may be that the subthalamo-ponto-cerebellar circuit may signal an “off-line” status to the cerebellum, notifying that currently the basal ganglia have withheld execution of motor programs so that the cerebellar internal models can be safely used for off-line mental simulation. Kishore and Popa consider this latter hypothesis highly speculative. They do not completely agree with the first hypothesis, observing that while it might be true for gaze control, axial muscle control, and background spinal circuits’ modulation, it might be not true for complex motor control of the limbs as well as for motor plan discrimination. This is therefore one example of topic where consensus cannot be reached yet and a topic that is critical for future research to explore.

Lago-Rodriguez and Galea agree with the Doya’s perspective and extend it by specifying the importance of the anatomical connections between cerebellum and posterior parietal cortex to motor control and adaptation. The authors review evidence suggesting that error-based motor learning occurs through multiple processes that are not all cerebellar-dependent (reinforcement learning/use-dependent learning/explicit strategies) and that require the involvement of a cortical-subcortical network including cerebellum, frontal cortex, and basal ganglia. This view is also in agreement with the evidence reviewed by Bostan and Strick and with the theoretical proposal of Houk. In addition, Lago-Rodriguez and Galea discuss recent data suggesting that cerebellar inhibition is important for implementing motor control, while the alleviation of cerebellar inhibition is associated with motor adaptation (see the paragraph below on “*Techniques for systems-level analysis of cerebellar functions*” for more details on the non-invasive brain stimulation techniques used by Lago-Rodriguez and Galea to study this effect). Caligiore et al. support this dual role of the cerebellum underlying how the increased motor adaptation due to an alleviation of cerebellar inhibition raises the question whether the cerebellum has an intrinsic role in motor adaptation or whether the cerebellum “opens” M1 to the influence of other networks (such as trans-cortical or fronto-striatal systems).

Miall presents neuroimaging evidence for the involvement of cerebro-cerebellar and cerebellar-basal ganglia interconnections in learning and long-term memory consolidation. On this basis, Miall supports the idea that the cerebellum employs a set of operating/learning principles that are replicated many times across the cerebellar cortex. Each replication has a specific function because it connects with specific brain regions. Miall gives three possible operating/learning principles, namely as a timing device, an associative learning network, and a short-term predictor, stressing the importance of the latter with respect to the other two. Not all the contributors agreed with this preference for the role of the cerebellum as a short-term predictor. For example, Houk supports the idea that all the three singular operating/learning principles are equally implemented by the cerebellum working in concert with cortical and basal ganglia areas. However, Miall also suggests that studies of learning from experience indicate that knowledge is consolidated in several cerebral sites outside the cerebellum after a time delay. Houk agrees with this view and suggests a possible mechanism underlying it which is based on the cerebello-cortical systems-level learning processes described in his entry. In particular, Houk describes an exportation mechanism whereby knowledge can be transferred from the cerebellum to the cerebral cortex through rehearsal or practice. This mechanism, which relies on Hebbian learning in cortex, tutored by input from the cerebellum, may be at the basis of the consolidation processes discussed by Miall. This hypothesis—which has parallels in the way other brain areas such as the hippocampus might “train” the cerebral cortex off-line [202, 203]—deserves future investigations.

### Cerebellar Systems-Level Mechanisms Underlying Dystonia and Parkinson’s Disease

Popa and Kishore describe how the altered interaction between cerebellar and fronto-striatal systems may play a role in movement disorders such as dystonia and Parkinson’s disease. In the same line, Caligiore et al. discuss how pathology in the interconnections between the cerebellum and the cerebral cortex and between the cerebellum and the basal ganglia reviewed by Bostan and Strick may contribute to the manifestation of resting tremor in Parkinson’s disease. There was a large agreement between the authors of the consensus paper about these systems-level perspectives to study dystonia and Parkinson’s disease. Caligiore et al. underline an interesting question raised by the paper of Popa and Kishore about to what extent the altered balance between activity in the basal ganglia and cerebellum in advanced Parkinson’s disease is caused by the primary pathology (i.e., dopamine depletion in the basal ganglia) or by a maladaptive compensation (e.g., increased activity in the cerebellum). Verschure et al. stress how the contribution of Popa and Kishore is very important as it spells out functional hypotheses on the systems-level interaction between cerebellum, basal ganglia, and cortex that can be translated into clinical applications. Verschure et al. propose that the nucleo-olivary inhibition discussed in their entry may have a critical role in explaining the hypotheses put forward by Popa and Kishore: the over-activation of the cerebellar cortex discussed by Popa and Kishore may result from excessive nucleo-olivary inhibition that, by silencing the inferior olive, increases the tonic level of activity in Purkinje cells [79]. This points to this specific pathway as a possible target for studies with animal models of Parkinson’s disease. It is also interesting to note a further link between the contribution of Popa and Kishore and the contributions of Lago-Rodríguez and Galea and of Miall: they all describe what appear to be congruent first- and second-order effects of the same basic principle of cerebellar control of the motor cortex. Namely, that cerebellar activity has an initial short-term effect on the control of neuronal activity in the motor cortex and a second long-term effect in controlling neuronal activity in motor cortex through plasticity.

Houk appreciates the “dimmer-switch” hypothesis proposed in [36, 183], further elaborated by Caligiore et al., underlining how it fits well with the DPM model of brain architecture outlined in his contribution. In addition, Houk proposes a possible systems-level mechanism within the DPM model to account for resting tremor. Using the DPM model, Houk predicts that in Parkinson’s disease, the loop through basal ganglia suffers from depleted dopamine and therefore learns goals poorly. As a consequence, it initiates actions pathologically in the positive feedback loop between the motor cortex and the cerebellar nucleus: instead of a normal motor command, what is initiated is an oscillation at the tremor frequency, and this oscillation is sustained and amplified by positive feedback. Purkinje cells in the cerebellar cortex have difficulty to refine this tentative action, so they occasionally just increase their discharge to force the bistable loop into its down state. This terminates the tremor completely. Then, after a while, the basal ganglia try again to initiate an action, and again, this results in an oscillation rather than in a proper action. This hypothesis suggests that tremor initiation may be a failed initiation of a voluntary action, which emerges as a tremor oscillation in the dopamine-depleted basal ganglia loop. This suggestion is at first surprising, given that the classical Parkinson’s tremor occurs at rest and disappears when patients make a voluntary action [204]. On the other hand, there is empirical support for such a mechanism: Hallett and colleagues [205] have shown that the pattern of alternating activity in agonist and antagonist muscles seen during Parkinson’s disease resting tremor strongly resembles the activity seen during voluntary flexion of the arm. They also found that in many patients with Parkinson’s disease, a single “beat of tremor” preceded voluntary movements, even when there was no clinically noticeable tremor. This suggests that (the initiation of) resting tremor and voluntary actions arise from similar oscillations in the motor cortex, which may explain why they do not occur simultaneously [36].

### Techniques for Systems-Level Analysis of Cerebellar Functions

The contributions of Popa and Kishore and of Lago-Rodríguez and Galea touch a field that is fundamental to the debate of this consensus paper: the study of systems-level interactions using non-invasive perturbation methods. The contribution by Popa and Kishore describes the first test of protocols of TMS applied to the cerebellum aimed at the alleviation of dyskinesia in PD patients. The success of such protocols is explained based on the modulation of motor cortex plasticity by the cerebellum. In the same line, Lago-Rodríguez and Galea review the effect of TMS and tDCS perturbations of the cerebello-cortical interactions both in control and learning during reaching tasks. The premise is that TMS in the cerebellum has an inhibitory effect on the M1—the so-called CBI. Thus, the main result reviewed is that CBI is lateralized stronger for the dominant hemisphere. Additionally, it is also highlighted that CBI decreases during motor adaptation. Regarding the interactions between cerebellum and basal ganglia, the authors sketch the methods that would allow the test of those interactions with non-invasive techniques. Interestingly, the data discussed by Miall in his contribution further support the analysis of the method discussed by Lago-Rodríguez and Galea. Moreover, the data discussed by Lago-Rodríguez and Galea, related to the critical contribution of cerebellar inhibition in motor control and adaptation, and the hypothesis supported by Verschure et al. about the gradual recruitment of the cerebellum through the regulation of the nucleo-olivary inhibition, reinforce reciprocally. Verschure et al. propose a mechanism (idiosyncratic of the olivo-cerebellar system) through which the amplitude of the modulations of the cerebellar output could be adjusted following contextual changes. Verschure et al. suggest that the sudden introduction of a perturbation in an adaptation protocol may cause a loss of certainty that would induce the cerebellum to decrease its anticipatory/predictive contribution to ongoing behavior. Then, a tempting hypothesis is that CBI can be a read-out of that contribution and its decrease a fingerprint of the ensuing modulation.

Most contributors of this paper agree that the involvement of trans-cortical and fronto-striatal systems to support the dual role of the cerebellum for motor control/adaptation (Lago-Rodriguez and Galea) and the interaction between the cerebello-fronto-striatal pathways (Popa and Kishore) and between subthalamo-ponto-cerebellar and cerebello-thalamo-cortical circuits (Caligiore et al.) is an important objective for further research and will likely require new network-based monitoring/therapeutic methods and systems-level computational models.

### Cortico-Subcortical Interactions Support Higher Cognitive Functions

Another emerging theme across several contributions of this consensus paper is that the systems-level view pursued here could be important to re-think the traditional hypotheses on the motor and especially the cognitive functions of the cerebellum in the light of the most recent evidence ([1, 2, 133]; see the recent consensus paper of Koziol and colleagues [51]). In this respect, Bostan and Strick highlight how the reciprocal anatomical loops between the cerebellum and the cerebral cortex involve both motor and non-motor cortical areas. In the same line, Miall underlines how the cortical-cerebellar network can be important for managing predictive mechanisms during language processing [124]. This claim is supported by empirical data showing that the right lateral cerebellum is connected with cortical language and higher cognitive areas such as the dorsolateral prefrontal cortex and the Broca’s area [1, 124].

This perspective suggests that higher level cognitive processes are not segregated within “higher-level cortical areas,” but they are realized in the same cortical-subcortical loops that support actions. In more details, cortical areas implement cognitive functions through mechanisms involving cerebellum and basal ganglia, and they depend on subcortical processes in order to execute many of their functions efficiently. In this way, higher-level functions of the cortex are expressed through its interactions with “lower level” systems which, in turn, are critical in modulating cortical functions [134]. This view contrasts the traditional distinction between brain areas which support higher cognition (mainly cortex) and brain areas which support action (mainly basal ganglia/cerebellum) and perception [135, 136]. This perspective is also supported by the embodied-grounded theories of cognition that are increasingly challenging traditional views of cognition by arguing that cognitive representations forming high-level knowledge are grounded in sensory and motor experiences rather than being processed in an amodal/abstract conceptual system [137–140]. An increasing amount of empirical evidence supports embodied cognition views. Examples include changes in motor behavior or perceptual experience as a result of semantic processing [140–150], or changes in categorization [151, 152] and mental manipulations of objects [153, 154], that reflect sensory and motor experiences (see [141] for a recent review).

Given the established empirical foundation, and the relatively underspecified theories to date, many researchers are extremely interested in embodied-grounded cognition but are clamoring for more specification about how the cortical-subcortical brain areas may be involved [139]. Embodied-cognition views are also supported by conceptual and computational models proposing specific computational mechanisms on how high-level cognition processes might strongly depend on processes implemented in brain sensorimotor areas. For example, an intriguing hypothesis is that one such cortico-cerebellar loop might permit the control of internal thought processes, reusing the same internal models and mechanisms that permit the cortico-cerebellar control of overt actions [142]. A related hypothesis is that the architecture of higher cognition and executive function is a sophistication of—and largely reuses—existing sensorimotor circuits and associated internal models, rather than relying on segregated neuronal circuits, but in an off-line (detached) mode [143]. In this perspective, the off-line re-enactment of action, perception, and emotional processes, which is based on internally generated (not stimulus-dependent) brain dynamics [198], realizes higher cognitive functions such as planning and long-term decisions and also entrains the online sensorimotor processes that ultimately realize the planned courses of actions [9, 144, 145]. Supporting this idea is the fact that the neuronal systems for mental imagery and motor preparation are closely related [20, 146–148, 153, 154]. The conceptual and computational models summarized here only grasp the surface of the cortical-subcortical processes that support higher cognition. The systems-level view of cerebellar functions presented in this article might be the basis of further theoretical, empirical, and modelling investigations addressing the problem of which brain structures and mechanisms underlie embodied-grounded cognition and emotional/motivational processes.

### Concluding Remarks

As this paper and especially the final discussion has highlighted, the integrative dynamics of the cerebello-basal ganglia-thalamo-cortical system is a vibrant and relatively new theme, which is already generating a variety of empirical studies, computational explorations, and theoretical hypotheses. While a definite consensus statement on how the cerebellum works in synergy with basal ganglia and cortical areas has not yet been reached, the contributions presented in this paper all recognize the central importance of studying cerebellar function from a wider, systems-level perspective that fully acknowledges its close interplay with different brain areas. The papers cover a broad spectrum of themes, some of which are relatively more consolidated, while others are more novel and even speculative, thus deserving future investigations. The combination and elaboration of the ideas presented here represent a first step towards a new consensus on the function of the cerebellum within larger brain networks and hold the promise to understand the cerebellar, basal ganglia, and cortical functional and learning processes in radically different ways.
